# Fluselenamyl: A Novel Benzoselenazole Derivative for PET Detection of Amyloid Plaques (Aβ) in Alzheimer’s Disease

**DOI:** 10.1038/srep35636

**Published:** 2016-11-02

**Authors:** G. S. M. Sundaram, Dhruva D. Dhavale, Julie L. Prior, Ping Yan, John Cirrito, Nigam P. Rath, Richard Laforest, Nigel J. Cairns, Jin-Moo Lee, Paul T. Kotzbauer, Vijay Sharma

**Affiliations:** 1 ICCE Institute, Molecular Imaging Center, Washington University School of Medicine, MO 63110, USA; 2 Mallinckrodt Institute of Radiology, Washington University School of Medicine, MO 63110, USA; 3 Department of Neurology, Washington University School of Medicine, MO 63110, USA; 4 Hope Center for Neurological Disorders, Washington University School of Medicine, MO 63110, USA; 5 Knight Alzheimer’s Disease Research Center, Washington University School of Medicine, MO 63110, USA; 6 Departments of Chemistry & Biochemistry, University of Missouri, St. Louis, MO 63121, USA; 7 Department of Pathology & Immunology, Washington University School of Medicine, MO 63110, USA; 8 Department of Biomedical Engineering, School of Engineering & Applied Science, Washington University, St. Louis, 63105, USA.

## Abstract

Fluselenamyl (5), a novel planar benzoselenazole shows traits desirable of enabling noninvasive imaging of Aβ pathophysiology *in vivo*; labeling of both diffuse (an earlier manifestation of neuritic plaques) and fibrillar plaques in Alzheimer’s disease (AD) brain sections, and remarkable specificity for mapping Aβ compared with biomarker proteins of other neurodegenerative diseases. Employing AD homogenates, [^18^F]-**9**, a PET tracer demonstrates superior (2–10 fold higher) binding affinity than approved FDA tracers, while also indicating binding to high affinity site on Aβ plaques. Pharmacokinetic studies indicate high initial influx of [^18^F]-**9** in normal mice brains accompanied by rapid clearance in the absence of targeted plaques. Following incubation in human serum, [^18^F]-**9** indicates presence of parental compound up to 3h thus indicating its stability. Furthermore, *in vitro* autoradiography studies of [^18^F]-**9** with AD brain tissue sections and *ex vivo* autoradiography studies in transgenic mouse brain sections show cortical Aβ binding, and a fair correlation with Aβ immunostaining. Finally, multiphoton- and microPET/CT imaging indicate its ability to penetrate brain and label parenchymal plaques in transgenic mice. Following further validation of its performance in other AD rodent models and nonhuman primates, Fluselenamyl could offer a platform technology for monitoring earliest stages of Aβ pathophysiology *in vivo*.

Alzheimer’s disease (AD) is the most frequent form of dementia which affects 24 million people worldwide, and also lacks effective therapeutic interventions^1^. Without successful treatment or prevention, the number of affected individuals can be expected to grow exponentially to 13–16 million in the United States and to >100 million globally by 2050. The failure of clinical drug trials to reverse clinical symptoms indicates that for a given treatment to be effective, it most likely needs to be prescribed at a preclinical stage before the symptomatic expression of the disease. Therefore, there is an urgent need to identify and validate biomarkers that are present at preclinical stages. Importantly, several biomarkers identified for diagnosis, staging, and assessment of therapeutic effects are (but not limited to): amyloid deposition, changes in CSF levels of tau, hyperphosphorylated tau (p-tau), or Aβ_1–42_, and reduced metabolism monitored via fluorodeoxyglucose (FDG) PET imaging^2^,^3^,^4^,^5^. While amyloid deposition and variations in CSF levels of tau and Aβ represent pathophysiological markers thus relevant for disease diagnosis, the reduced metabolism (FDGPET) or atrophy (MRI) demonstrate topographic markers indicating a progression of the disease. Furthermore, literature precedents of last decade indicate that AD pathological changes (Aβ deposition and NFT formation) occur years prior to onset of symptoms^6^. For diagnosis of AD, several PET radiopharmaceuticals targeting Aβ deposition, such as, [^11^C]-PiB^7^, [^18^F]-FDDNP^8^, [^11^C]-SB-13^9^, and [^18^F]-AV-45^10^, [^18^F]-AZD4694^11^,^12^ have been investigated in humans. In addition, [^125^I/^131^I]-TZDM, [^125^I]-IMPY, [^123^I]-DRM106 and [^123^I]-ABC577, have also been investigated for SPECT applications^13^,^14^,^15^. While [^11^C]-PiB has been most intensely studied, [^18^F]-AV-45^16^, [^18^F]-Flutemetamol (Vizamyl)^17^,^18^ and [^18^F]-Florbetaben (Neuraceq™)^19^,^20^,^21^ have been recently approved by FDA for Aβ imaging. Importantly, both [^18^F]-AV-45^22^ and [^11^C]-PiB show promising results in humans and excellent correlation with FDG^7^. Recent investigations examining [^3^H]-PiB and [^18^F]-AV-45^23^ binding to AD homogenates also indicate multiple binding sites on Aβ^24^ thus mandating development of new tracers to study Aβ pathophysiology. To further supplement the existing armamentarium of FDA approved Aβ imaging agents, earlier we have shown that a heterocyclic fluorescent molecule is capable of traversing the BBB to label Aβ plaques in brains of APP^+/−^/PS1^+/−^ mice and also indicates sensitivity for detecting diffuse plaques in autopsy confirmed AD human tissues^25^,^26^. Although the PET counterpart showed high first pass extraction into the brains of normal mice and Aβ labeling in brain frontal cortex of APP^+/−^/PS1^+/−^ mice, its binding affinity to AD homogenates was 5–10 fold inferior compared with two FDA approved agents^27^. Importantly, this template scaffold of our first generation agent comprised a benzothiazole moiety, a pharmacologically active constituent with widespread medicinal chemistry applications. Compared with benzothiazole derivatives, investigations on biological activity of benzoselenazoles have not gained much attention primarily due to a lack of convenient and cost-efficient chemical methodologies^28^. To our knowledge, benzoselenazoles derivatives represent an entirely novel class of Aβ diagnostic agents. Herein, we report synthesis, characterization and crystal structure of (Z)-5-(2-(5-(2-fluoroethoxy)benzo[d][1,3]selenazol-2-yl)vinyl)-N,N-dimethylpyrimidin-2-amine (Fluselenamyl: **5)**, F-18 labeled radiotracer ([^18^F]**-9**), and perform its preclinical validation to evaluate its potential to serve as an Aβ-targeted PET radiopharmaceutical for monitoring plaque burden in AD. Fluselenamyl demonstrates potent binding to Aβ fibrils, autopsy confirmed AD homogenates, traverses the blood brain barrier (BBB) to detect Aβ plaques in a transgenic mice model, and is highly specific for probing Aβ plaques in AD.

## Materials and Methods

Material details, organic synthesis procedures, analytical characterization of all intermediates including the final Fluselenamyl (1, 2, 3, 4, 5, 6, 7, and 8) and radiochemistry for synthesis of the PET tracer [^18^F]**-9** are described in supporting information. Additionally, X-ray crystallographic details for **5**; Aβ binding assays (Fibrils and AD homogenates) using **5** and [^18^F]**-9**; histochemical staining of **5** with transgenic mice brain cross sections, and AD brain sections; human serum stability of [^18^F]**-9**; autoradiography of [^18^F]**-9** with AD human brain sections and ex vivo brain sections of APP/PS1 and WT mice biodistribution studies of [^18^F]**-9** in FVB mice; multiphoton imaging using **5** in transgenic mice, and finally microPET/CT imaging details of [^18^F]**-9** in age-matched transgenic mice and their WT counterparts are also included in the supporting information. While postmortem brain tissues from autopsy-confirmed AD patients and their approximate age-matched healthy controls were obtained through the Knight Alzheimer’s Disease Research Center (ADRC) Washington University School of Medicine, and processed according to a protocol approved by institutional ADRC executive committees, the animal procedures were approved by the Washington University Animal Studies Committee (Protocols #20150147, PI: Sharma; protocol #20140182, PI: Jin-Moo Lee). All methods were carried out on animals and human tissues in “accordance” with the approved guidelines.

## Results and Discussion

**Figure 1:**
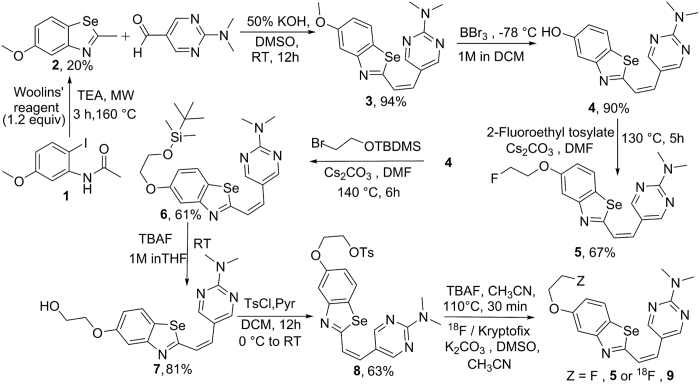
Chemical synthesis of Fluselenamyl **5** and [^18^F]-**9 (**PET tracer).

**Figure 2:**
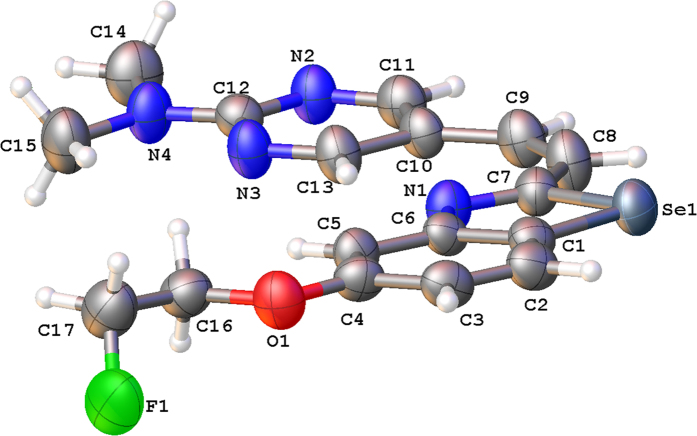
The projection view of **5** showing crystallographic numbering scheme. Atoms are represented by thermal ellipsoids corresponding to 30% probability.

Our strategic design for obtaining a second generation Aβ-targeted agent involved five functional components described earlier^25^. Incorporating those characteristic features into a template scaffold, the Aβ targeted probe **5** was synthesized as shown (Fig. 1; synthetic chemistry details are in supporting information). For synthesis, 5-methoxy-2-methylbenzo[d][1,3]selenazole **2** was obtained using literature procedures from N-(acetyl)benzoyl-2-iodoaniline **1**, using Woolins reagent under microwave conditions^29^ and condensed with 2-(dimethylamino)pyrimidine-5-carbaldehyde in an aqueous potassium hydroxide (50%) solution dissolved in DMSO to obtain **3.** Following purification, **3** was demethylated in the presence of BBr_3_ to yield the phenolic derivative **4**. Finally, **4** was alkylated with 2-fluoroethyl-4-methylbenzene sulfonate (prepared using literature procedure^30^) in the presence of cesium carbonate to obtain **5**. Alternatively, **4** was treated with (2-bromoethoxy)(tert-butyl)dimethylsilane in the presence of cesium carbonate to obtain (Z)-5-(2-(5-(2-((tert-butyldimethylsilyl)oxy)ethoxy)benzo[d][1,3]selenazol-2-yl)vinyl)-N,N-dimethylpyrimidin-2-amine (**6)**. Upon treatment with TBAF, its corresponding deprotected alcohol **7** was obtained. Following treatment of **7** with tosyl-chloride, **8** the precursor ligand for synthesis of the PET tracer was obtained. Finally, **8** was also treated with TBAF to obtain **5** via nucleophilic displacement. All intermediates **1**, **2**, **3**, **4**, **6**, **7, 8** and the final compound **5** were characterized via standard analytical methods. Additionally, **5** was also analyzed for uniformity and purity, on a semi-preparative C-18 column (Phenomenex), using an HPLC system. Compound **5** eluted as a single chemical entity, with a retention time of 10.5 minutes thus indicating purity of the molecule. For determination of the solid-state structure, crystals suitable for X-ray analysis were obtained via a vapor diffusion method, involving slow diffusion of methanol into a DCM solution containing 1% ACN of **5**. The benzoselenazole derivative **5** crystallized in a monoclinic space group P 2_1_/n. The ORTEP drawing showing the crystallographic numbering scheme for **5** is illustrated in Fig. 2. The crystal structure of **5** shows a planar molecule with the F atom displaced from the mean plane by 1.005 Å (mean plane deviation for the molecule except F = 0.048 Å). The heterocyclic molecule **5** indicates presence of both inter and intra-molecular hydrogen bonding (SI, Fig. 1). Noticeably, H13 forms intra-molecular H bond to N1 at 2.22 Å thus comprising a bond angle of C13-H13-N1 = 147°. Additionally, two intermolecular hydrogen bonds involve the N atoms N2 from the ring and the solvent N, N1s [C3-H3….N2 (1.5-x, y-0.5, z-0.5) = 2.61 Å and the bond angle around H3 = 164.5°; C15-H15a…N1s (1+x, y, z) = 2.68 Å and the angle around H15a = 170.5°]. While search of the Cambridge Crystallographic database does not show organic scaffolds sharing a similar arrangement of atoms around Se in the molecule, the closest chemical structure for comparative analysis remains the first generation Aβ targeted molecule reported earlier^25^. Importantly, the chemical structures of **5** and its first generation benzothiazole derivative counterpart (C_18_H_18_FN_3_OS) are identical except that Se is swapped with S (while the six membered aromatic ring of **5** possesses 2Ns, the aromatic ring of the benzothiazole derivative contains only one N atom). Therefore, Se-C interatomic distances (1.868(7) and 1.917(7) Å) in **5** are larger compared to the S-C distance (1.728(2) and 1.762(2) Å) in earlier reported benzothiazole derivative thus consistent with a larger size of the selenium atom. Furthermore, the bond angle around the Se atom of **5** is also significantly narrower than that of benzothiazole derivative (84.6° vs 89.1°). Overall, comparative analysis indicate both **5** and its first generation counterpart^25^ share planar geometry with the F atoms displaying out of plane deviation. Finally, NMR spectral data of **5** was also consistent with the crystal structure thus indicating the presence of identical structures both in solid and solution state (*J*_H-H_ = 7.4 Hz; alkene protons indicating the presence of Z isomer) and the results are in accord with the first generation Aβ agent^25^.

For assessing the ability of **5** to bind Aβ plaques; preliminary binding assays with preformed Aβ_1–42_ fibrils were performed in PBS. Following excitation at 392 nm, fluorescence spectrum of **5** recorded in PBS containing 1% ethanol showed a broad emission peak at 450–540 nm with E_max_ at 490 nm. Upon incubation with preformed Aβ (1–42) aggregates, the peak (490 nm) showed remarkable enhancement in fluorescence indicating binding to Aβ aggregates, similar to enhancement in fluorescence of thioflavin T in PBS (a positive control; data not shown). Additionally, it is also noteworthy that no fluorescence was observed using Aβ aggregates alone in PBS following excitation at 392 nm (a negative control). Preliminary binding assays of **5** with preformed Aβ_1–42_ aggregates (using a single site binding model) indicated saturable binding with a K_d_ = 1.58 ± 0.05 nM (SI, Fig. 2).

**Figure 3:**
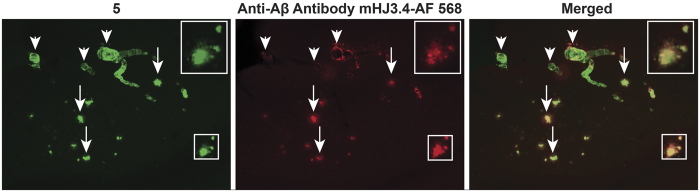
Staining of brain hippocampus tissue sections from APP^+/−^/PS1^+/−^ 10 months old mice using **5** (100 nM) or immunostained with mouse monoclonal antibody (mHJ3.4) conjugated to Alexa 568 (positive control). Arrows indicate labeling of Aβ plaques (arrows, extracellular Aβ; arrow head, Cerebral Amyloid Angiopathy (CAA). The slides were analyzed Nikon Eclipse E800 epifluorescence microscope. Magnification: 10X; Top Inset; 20X.

Aβ imaging ligands and disease-modifying therapeutics have been investigated using APP^+/−^/PS1^+/−^ transgenic mice models^31^,^32^. Further, we assessed the ability of **5** to stain *ex vivo* brain sections (50 μm) of age-matched APP^+/−^/PS1^+/−^ mice and their WT (BL/6) counterparts, using established procedures^33^. As a positive control, anti-Aβ monoclonal antibody (mHJ3.4 conjugated to Alexa Fluor 568) was used^34^. Brain sections of 10 month old APP^+/−^/PS1^+/−^ mice, using mHJ3.4-AF568 conjugate showed distinct staining of Aβ (Fig. 3) compared with none in WT counterparts (SI, Fig. 3). Similarly, **5** (100 nM) demonstrated abundant staining of Aβ plaques in the hippocampus brain sections in APP^+/−^/PS1^+/−^ mice. By comparison, **5** indicated no staining in 10 months old WT mice (SI, Fig. 3) thus indicating its target specificity. While nearly 1:1 correlation was apparent for Aβ extracellular plaques (Fig. 3; right panel; arrows), 5 also demonstrated substantially higher sensitivity for labeling CAA compared with anti Aβ antibody thus consistent with slight variations in staining patterns observed between a small organic molecule and a large antibody^25^.

**Figure 4:**
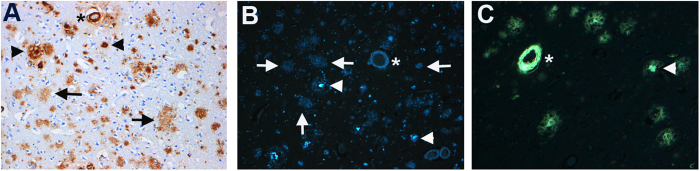
Binding of anti-Aβ antibody (10D5, Eli Lilly, (**A)**), **5** (**B**), and thioflavin S (**C**) to Aβ plaques in AD brain near/adjacent tissue sections. Amyloid in postmortem frontal lobe of a 90-year-old male. Magnification: 200X. (**A**): diffuse Aβ plaques and an arteriole with Aβ deposits (cerebral amyloid angiopathy); 10D5 immunohistochemistry. (**B**): section stained with **5** containing the same arteriole as in (**A**). There are numerous diffuse Aβ plaques (arrows) and compact plaques (arrow head) and the vessel is also stained. (**C**): Thioflavin S reveals amyloid in blood vessels and compact plaques (arrow head). The same blood vessel (asterisk) is labeled in (**A**–**C**). Similar results were obtained with more than three independent experiments.

To further assess ability of the **5** to label Aβ plaques in human brain, staining experiments were also performed with postmortem tissues from clinically-characterized AD patients^35^,^36^ As a positive control, a highly specific anti-Aβ antibody (10D5, Eli Lilly, Indianapolis, IN) was used to ascertain the presence of Aβ plaques (Fig. 4A), using well-established procedures for assessment of Aβ plaques in postmortem brain^37^. Importantly, **5** (2 μM) demonstrated strikingly distinct labeling of Aβ plaques in the tissue sections of frontal lobe of a 90-year-old male with AD (Fig. 4B) and absence of Aβ plaques in normal controls (SI, Fig. 4A,D), therefore indicating target specificity. Noticeably, **5** also indicated proficient and distinct labeling of plaque and leptomeningeal vessels (cerebral amyloid angiopathy; CAA) (Fig. 4B). These data are consistent with other unlabeled counterparts of FDA approved PET agents^38^. Furthermore, thioflavin S, an amyloid staining dye showed staining of amyloid in the blood vessel (CAA) and indicated only weak staining of diffuse plaques (Fig. 4C). Importantly, **5** demonstrated labeling of numerous diffuse Aβ plaques (Fig. 4B) and specificity for Aβ plaques in AD compared with biomarkers of other neurodegenerative diseases (SI, Fig. 4A,B). This high specificity of **5** for Aβ could be attributed to presence of a benzoselenazole ring within Fluselenamyl. Overall, the ability and sensitivity of **5** to detect diffuse plaques and specificity for AD could represent an important advancement to enable PET imaging of mildly demented individuals, prior to onset of symptoms^6^.

For performing additional bioassays and correlating Aβ binding data of **5** obtained via fluorescence assay, the PET counterpart [^18^F]-**9** was synthesized via standard nucleophilic substitution, employing 2,2,2-kryptofix/^18^F and tosylate analog **8**. Following reaction and Sep-Pak treatment to separate free fluoride, the crude mixture was purified on a C-18 column, using a radio-HPLC system, with an overall radio-chemical yield of 35% (radiochemical purity >99%; specific activity (1700–2000 Ci/mmol). Furthermore, [^18^F]-**9** was also characterized by spiking with an analytically characterized sample of an unlabeled counterpart **5** (SI Fig. 5), prior to injection on the radio-HPLC. The fraction eluting at R_t_ = 10.5 min was collected, concentrated, and resuspended in PBS/ethanol (95/5) for all radiotracer bioassays.

**Figure 5:**
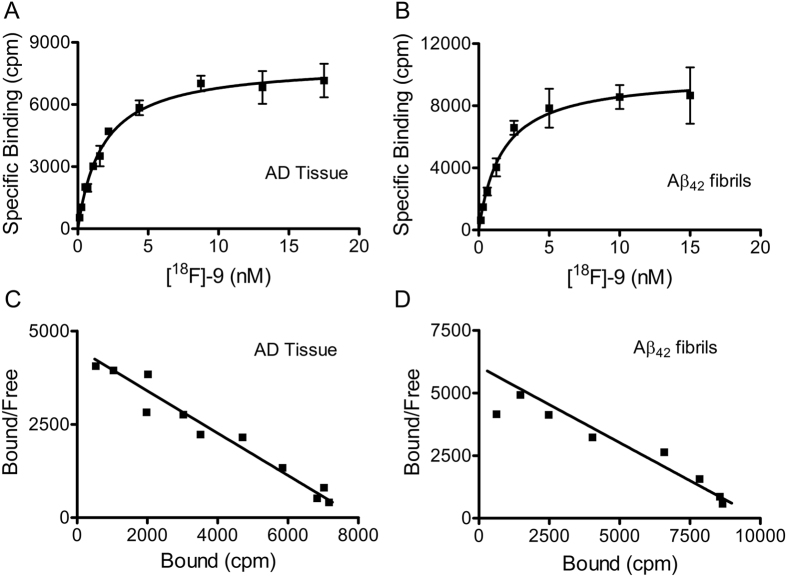
Binding of [^18^F]-**9** with AD homogenates (**A**,**C**) and Aβ_1-42_ fibrils (**B**,**D**). **Aβ**_**1-42**_fibrils and AD homogenates were incubated with increasing concentrations of [^18^F]-**9**. Representative plots of specific binding versus [^18^F]-**9** concentration are shown for AD homogenates in (**A**) and Aβ fibrils in (**B**). Data points represent mean +/− standard deviations (n = 3). The data was analyzed by curve fitting to a one-site binding model using a nonlinear regression. Scatchard plots of binding are shown for AD homogenates (**C**) and Aβ_1-42_ fibrils (**D**) and are consistent with one-site binding model. Similar results were obtained in two independent experiments. Fluorescence binding assays of **5** with Aβ_1-42_ fibrils also indicated a saturable specific binding with a K_d_  = 1.58 ± 0.05 nM (SI, Fig. 2).

For assessing the ability of [^18^F]-**9** to bind Aβ plaques; binding assays with either AD homogenates or preformed Aβ_1–42_ aggregates were performed in 30 mM Tris, pH 7.4 buffer supplemented with 0.1% BSA^39^. Nonspecific binding was determined in the presence of **5** (1 μM) as a competitor. Overall, the binding assay of [^18^F]-**9** with AD homogenates and Aβ_1–42_ fibrils (Fig. 5A–D), indicates a saturable specific binding with K_d_ = 1.7 nM (B_max_ = 546 pmol/g wet wt.) and 1.6 nM (B_max_ = 1.3 pmol/nmol), respectively (Fig. 5AB). Scatchard plots of the binding data indicate that [^18^F]-**9** binds to a single high affinity site on AD homogenates and Aβ_1–42_ fibrils (Fig. 5C,D). Importantly, the binding affinity of [^18^F]-**9** with autopsy confirmed AD homogenates is significantly superior to that of other FDA approved Aβ-targeted probes ([^18^F]-Florbetaben, 16 nM^40^; [^18^F]-AV-45, 3.7 nM^41^; [^18^F]-Flutemetamol, 6.7 nM)^42^. Previously, the incorporation of pyrimidine ring into benzothiazole has been shown to decrease binding affinity of the molecule to Aβ fibrils^43^. However, the design of Fluselenamyl includes an incorporation of pyrimidine ring into benzoselenazole, wherein the atomic radius of selenium (115 pm) is much larger than that of sulfur atom (100 pm) present within benzothiazole^43^, resulting in longer bond lengths involving selenium with neighboring atoms of the 5-membered ring (as evident from crystal structure data of **5**), and the presence of an additional double bond between two ring systems (benzoselenazole and pyrimidine ring) thus generating a relatively better flow of electrons consistent with planarity of the molecule and observed strong fluorescence enhancement of **5** upon binding to Aβ compared with analogues of PiB^42^. Additionally, the incorporation of dimethylamino group into the pyridine ring has been shown to promote selectivity for Aβ^44^. Therefore, the higher binding affinity of **5** to Aβ plaques could be attributed to these combined variations in the scaffold.

**Figure 6:**
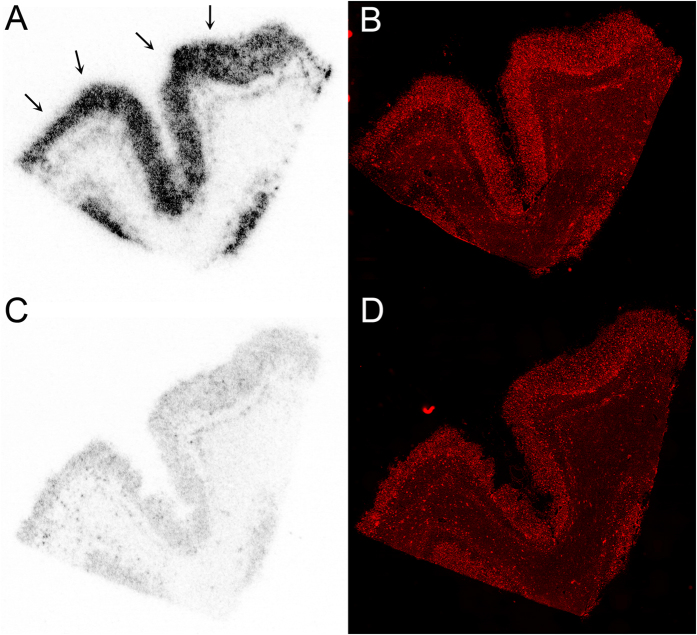
Autoradiography images of [^18^F]-**9** binding in an AD frontal cortex section following incubation with either [^18^F]-**9** (2 nM) alone (**A**) or in the presence of **5** (**1 **μM, **C**). Fluorescent immunostaining of sections (**A**) and (**C**) with an anti-Aβ antibody conjugate is shown in (**B**) and (**D**), respectively. The autoradiography images demonstrate laminar distribution of [^18^F]-**9** binding in cortex, which correlates with the distribution of Aβ plaques detected by fluorescent immunostaining, and binding of [^18^F]-**9** is inhibited by excess cold ligand **5** (**1 **μM, **C**).

Literature precedents indicate the presence of at least three different binding sites, characterized as BS1, BS2 and BS3 on the Aβ fibrils *in vitro*^24^ and extracts from AD homogenates^23^. A recent binding study using homogenates from AD confirmed human tissues and their control counterparts also indicates the presence of multiple binding site models for the amyloid tracers^23^, wherein [^18^F]-AV-45 and [^11^C]-PiB have been shown to bind to two different binding sites, a high-affinity site (visualized by PET) and a low affinity site. Additionally, while BF-227 shows binding to BS3, the FDDNP has been postulated to bind only to BS2^23^. Overall, these investigations indicate that different PET tracers may be beneficial to better understand Aβ pathophysiology *in vivo*. To assess the binding site targeted by [^18^F]-**9** on AD homogenates, binding assays were also performed in the presence of unlabeled analytically characterized samples of PiB, IMPY, and Chrysamine G. While no displacement was observed with Chrysamine G, significant displacement of [^18^F]-**9** specific binding was observed in the presence of unlabeled PiB and IMPY, suggesting that radiotracer [^18^F]-**9** binds to the same high affinity site on AD homogenates. This is further supported by the observation that the K_i_ values obtained from analysis of the competition assays (K_i_ = 4.9 nM for PiB, K_i_ = 6.3 nM for IMPY) are also consistent with previously reported K_d_ values obtained in assays measuring direct binding of these tracers to AD homogenates. Further, to evaluate ability of [^18^F]-**9** for labeling Aβ in autopsy confirmed human brain sections, autoradiography and immunohistochemical correlations were also performed. Following incubation of AD frontal cortex sections (12 μm) with [^18^F]-**9** (2 nM) for 60 min, the agent showed labeling of cortical Aβ plaques and the binding was inhibited upon incubation in the presence of **5** (1 μM) (Fig. 6A,C). These data indicate sensitivity and specificity of the [^18^F]-**9**. Additionally, immunohistochemical staining of these sections using anti- Aβ-antibody indicated the presence of Aβ plaques in the cortex of these sections (Fig. 6B,D) thus demonstrating excellent correlation of immunohistochemical staining data with that of autoradiography data.

For biomedical imaging applications, the signal is a net function of target/background ratio. In PET imaging, this signal results from detection of γ-photons arising from annihilation events of positrons and imaging resolution is also dependent upon energy associated with a given radionuclide. Resultant radioactive metabolites could also contribute to nonspecific binding or compete with the parental tracer for binding to the target. Therefore, it is also important to investigate metabolic stability of the radiotracer to explicitly confirm whether or not biochemical targeting profiles of a given tracer are driven by a parental molecular imaging probe. Importantly, both [^11^C]-PiB and [^18^F]-AV-45 have demonstrated low biological half-lives in serum; while metabolites of [^11^C]-PiB have been shown to be polar and thus postulated to not penetrate the brain^45^, two metabolites of [^18^F]-AV-45 (desmethylated, 4.5% ID/g; acetylated analogue, 3.3% ID/g at 2 min in normal mice)^10^ have been shown to permeate the brain and thus could potentially contribute nonspecific interaction and complicate image analysis. To perform preliminary evaluation of biological half-life, [^18^F]-**9** was also incubated at 37 °C in human serum as a function of time, and aliquots were analyzed on radio-HPLC. The presence of a single radio-peak indicated presence of parental tracer up to 3 h (SI Fig. 6).

**Figure 7:**
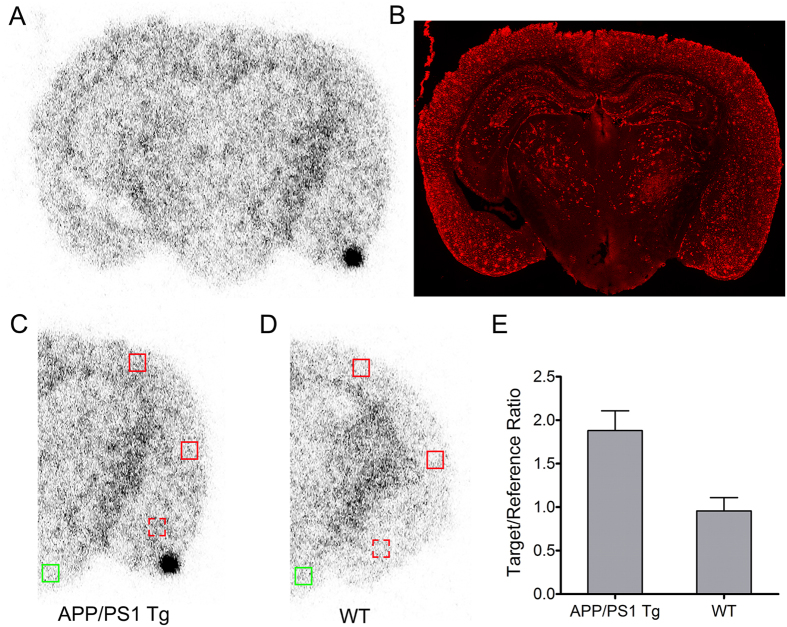
*Ex vivo* autoradiography images of 24-mo APP/PS1 Tg and WT control mouse after injection with [^18^F]-9. (**A**) Coronal autoradiography image obtained at 30 min post-injection of [^18^F]-**9** in the 24 months APP/PS1 Tg mouse. (**B**) Immunofluorescence staining of the same section with monoclonal anti-Aβ antibody to assess plaque density. (**C**,**D**) Count intensities were determined in three target ROIs (red boxes) for APP/PS1 Tg (**C**) and WT control (**D**), based on high Aβ plaque density in the immunostained APP/PS1 Tg section. Solid red boxes represent the locations of target ROIs in cortex, while dashed red boxes represent the locations of target ROIs in amygdala. Count intensities were also measured in a reference ROI (hypothalamus) with low plaque density, represented by green boxes. (**E**) Levels of tracer activity were analyzed by comparing the target/reference count intensity ratios for APP/PS1 Tg and WT control mice (mean + SD, n = 3).

To further assess whether or not PET counterpart [^18^F]-**9** administered at tracer concentrations relevant for nuclear imaging demonstrates optimal kinetics (signal/noise ratios) to enable brain imaging *in vivo*, quantitative biodistribution studies in normal mice were performed. Uptake in brain and other critical organs was analyzed in terms of percent injected dose per gram of the tissue (%ID/g) SI Table 1. For *in vivo* imaging of Aβ plaques, the basic pharmacokinetic model in normal brains involves a high initial penetration of the agent, accompanied by facile clearance due to lack of a binding target. Preliminary biodistribution studies (SI, Table 1) with HPLC purified [^18^F]-**9** in normal mice show transient brain uptake values of 8.86 ± 0.32% ID/g and 1.66 ± 0.01% ID/g, at 2 min and 120 min post tail-vein injection, respectively, thus providing a 2 min/120 min clearance a ratio of 5.33. For comparison, brain uptake ratios of [^18^F]-AV-45 (2 min/2 h) and [^18^F]-Florbetaben (2 min/4 h) in normal mice are 4.07 (%ID/g (brain): 2 min: 7.33 ± 1.54; 2 h: 1.80 ± 0.07; 7.33/1.80; 4.07)^10^ and 5.0 (%ID/g (brain): 2 min: 4.77; 4 h: 0.95; 4.77/0.95; 5.02)^46^, respectively. Therefore, the brain uptake clearance ratio of [^18^F]-**9** (2 min/120 min) is 1.3-fold superior to [^18^F]-AV-45 and is comparable to that of [^18^F]-Florbetaben in healthy mice. For an agent to be able to serve as an Aβ-imaging agent, literature precedents indicate that brain uptake ratio (%ID/g; the earliest time–point; 2–5 min to that of the latest time point; typically for carbon-11; 30–60 min; and F-18, 2 h) of 3.5 or above could be considered as a benchmark for ability of a given agent to cross the blood-brain barrier^38^. Additionally, the brain uptake of a given imaging agent is also a net function of several components, such as cerebral regional blood flow, BBB permeability, plasma radiotracer concentration, and free fractions of the radiotracer in plasma and in the brain. Furthermore, the lipophilicity of a given compound also reflects a critical physicochemical trait for neuroimaging radiotracers due to its direct relationship to membrane permeability, solubility in water, and entropic contribution to binding. Literature precedents indicate that lipophilic drugs readily cross the BBB, although other chemical characteristics, including the number of hydrogen bonds, molecular weight, polar surface area and molecular size are also known to be critical traits for passive transport. Lipophilicity measured via (log *P*_OCT_), the octanol/water partition coefficient for non-ionized molecules serves as a good indicator of a molecule to permeate brain and molecules possessing log *P* values of 0.9 and 3.0 have been shown to cross the BBB^47^. Conversely, radiotracers that are too lipophilic can also bind plasma proteins, undergo fast metabolism, while also contributing to high nonspecific binding, such as white matter. [^18^F]-**9** demonstrates a log *P* value of 1.28 which is similar to that of [^11^C]-PiB (1.3) but considerably lower than that of [^18^F]-AV-45 (2.4), and [^18^F]-Florbetaben (3.22). While the clearance ratio of 5.33 (%ID/g; 2 min/120 min; SI Table 1) provides evidence for the ability of [^18^F]-**9** to traverse the BBB *in vivo*, the log *P* value of 1.28 could also lead to a low nonspecific interaction with white matter. Additionally, the agent [^18^F]-**9** excreted from other critical organs over 2 h (SI, Table 1); a critical factor likely to result in favorable dosimetry; although slight defluorination as a function of time is also evident from bone accumulation; yet consistent with pharmacokinetic profiles of other FDA approved agents^10^,^17^,^18^. For assessing directly the ability of radiotracer to traverse the BBB, [^18^F]-**9** (170 μCi) was injected into a 24 months old APP/PS1 transgenic mouse and its age-matched WT counterpart, and 30 min post tail-vein injection, the brains were removed, frozen, and sectioned for autoradiography (Fig. 7A–E). Autoradiograms of the brain sections showed labeling of plaques in cortical regions, in addition to probable off-target binding in white matter (Fig. 7A). The labeling of plaques was confirmed by staining with anti-Aβ monoclonal antibody (mHJ3.4 conjugated to Alexa Fluor 568, Fig. 7B) and data show fair correlation with a caveat of some off-target binding attributed to differences between targeting profiles of a small organic molecule versus the large highly specific monoclonal antibody (Fig. 7A,B). For ROI analysis of the PET signal in brain section autoradiograms of transgenic mouse (Fig. 7A) and its WT counterpart (Fig. 7D), we used measured count densities in cortex and amygdala target regions and compared them with that of hypothalamus as reference region with a low plaque density as determined by Aβ immunostaining (Fig. 7B). The target/reference ratio (mean ± SD) in the transgenic mouse was 1.88 ± 0.22 compared to 0.96 ± 0.12 in the WT control mouse (Fig. 7E). Since brain stem was also devoid of plaques, we also analyzed the above target regions using brain stem as a reference region, and observed target/reference value in the transgenic to be 0.89 ± 0.1 compared to 0.45 ± 0.07 in the WT mouse.

**Figure 8:**
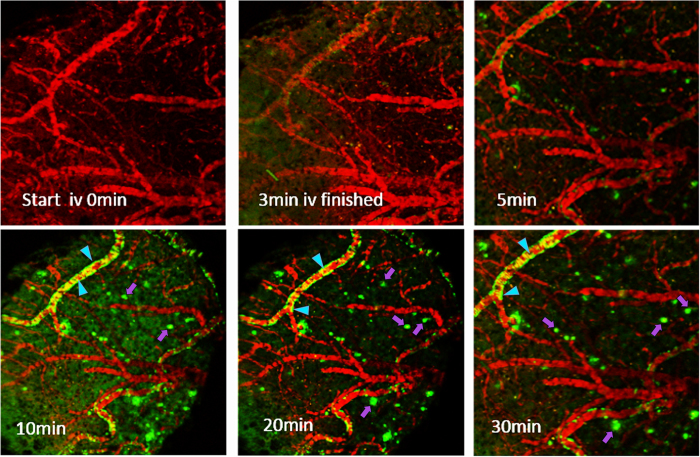
Real time multiphoton imaging of **5** in brains of APP ^+/−^/PS1^+/−^ transgenic mice: Following demarcation of blood vessels with dextran-Texas Red, **5** (2 mg/kg) was intravenously injected. A z-stack image series was acquired using an LSM 510META NLO microscope (Carl-Zeiss Inc). While arrowhead indicates vascular Aβ, the arrow shows parenchymal plaques.

For assessing viability of molecules as imaging probes *in vivo*^48^, various imaging modalities, such as nuclear imaging (PET/SPECT), optical imaging, and MRI have been used to investigate simultaneously distribution kinetics and target-receptor specificity. While the resolution of PET and MRI allow *in vivo* imaging at relatively moderate resolution, the multiphoton microscopy enables evaluation of kinetics at a sub-micrometer resolution^49^. Therefore, this technique enables characterization of probes in small animal models at a significantly high spatial and temporal resolution^49^. The generation of various transgenic (tg) animals overexpressing mutant human APP and/or PS1 and PS2, tg APP mice expressing ApoE isoforms including those for Tau offer attractive models for unravelling biochemical pathways prevalent in pathophysiology of AD^50^. Of note, compared to single transgenic animal models (APP or PS1), co-expression of PS1 with APP exhibits robust deposition of Aβ months earlier than APP tg mice alone. Literature precedents indicate Aβ deposition by 2 months of age, with gradual quantitative increase at six months^55^. Therefore APP/PS1 mice offers interesting models for evaluation of therapeutics and validation of imaging probes *in vivo*^25^. For assessing the ability of **5** to penetrate the BBB, label Aβ parenchymal plaques, and simultaneously interrogate the pharmacokinetic profiles from nearby brain regions, direct real-time imaging was performed in transgenic APP^+/−^/PS1^+/−^ mice. Prior to imaging, dextran-Texas Red conjugate (33 mg/kg; dissolved in PBS to mark the blood vessels) and **5** (2 mg/kg; dissolved in 20% DMSO in propylene glycol^56^) were intravenously administered to anesthetized APP^+/−^/PS1^+/−^ mice (with cranial windows; supporting information). Following injection, 3D volumes were acquired (by collecting a stack of x-y sections from surface of the thinned skull to 100 μm deep into the cortex). Compared with barely detectable auto-fluorescence levels prior to imaging transgenic APP^+/−^/PS1^+/−^ mice (Fig. 8, first panel), bright fluorescence appeared almost instantaneously following administration of **5**. Fluorescence first appeared in large and small blood vessels within the brain. Within minutes (10 min), the brain parenchyma was uniformly bright in fluorescence, and Aβ deposits were labeled. While the complete labeling of CAA occurred instantaneously following injection, the labeling of parenchymal plaques peaked at approximately 10 min. Overall, these data demonstrate in real time that **5** permeates the brain rapidly, and visualizes parenchymal Aβ plaques (Fig. 8).

**Figure 9:**
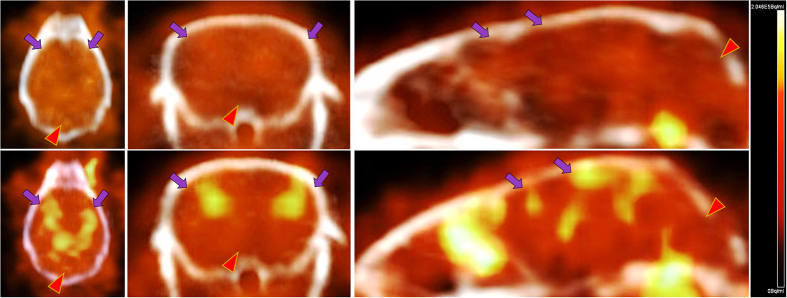
MicroPET/CT Imaging. APP/PS1 and WT mice (**15** months old; n = 3; closely age-matched) were injected intravenously with HPLC-purified [^18^F]-**9** (5.032 MBq). Representative PET static images of brain (Coronal, Axial, and Sagittal View) were obtained from 15–30 min post intravenous injection, and co-registered with CT for an anatomical reference. The scale shows a range (Min-Max) of 0–2.046 × 10^5 ^Bq/mL. Bottom: APP/PS1 mouse, Top. WT mouse. While arrow indicates cortex, the arrowhead depicts cerebellum; Note higher retention of [^18^F]-**9** in the brains of APP/PS1 (bottom) compared with WT counterpart (top).

**Figure 10:**
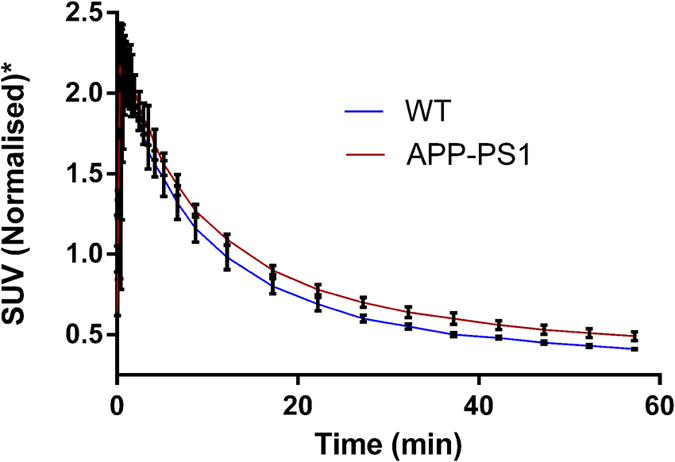
Dynamic PET scans were performed for 60 min following injection of [^18^F]-9 (5.032 MBq), and TAC (time activity curve) represent whole brain radioactivity post-intravenous injection of [^18^F]-9 in age-matched APP/PS1 and BL6 mice. The data were normalized to average count between 30 sec and 3 min time points.

To directly access the potential of [^18^F]-**9** to bind Aβ plaques *in vivo*, we performed microPET/CT imaging in age-matched APP^+/−^/PS1^+/−^ mice (n = 3) compared their WT counterparts, 5 min-2 h post tail-vein injection. For any given agent to serve as an Aβ-targeted agent, a pharmacokinetic model would involve an initial high and equal influx of the tracer into the brains of transgenic and WT mice, followed by clearance of the radiotracer from brains of WT mice thus demonstrating differential retention in regions of brains, as the agent binds to Aβ plaques in transgenic mice. Indeed, the [^18^F]-**9** demonstrates a 1.2-folds (p < 0.05) higher retention in transgenic mice brains consistent with its binding to Aβ plaques, while exhibiting clearance of the unbound tracer from brains of WT counterparts (Fig. 9). Time activity curves (TAC) also indicated higher retention of activity within transgenic mice brains compared with their WT counterparts (Fig. 10). Additionally, ROI (cortical regions) were also analyzed using linearized method with a reference region (cerebellum) via Reference Logan plot (RefLogan), and distribution volume ratios (DVR) were obtained. The analysis indicated a statistically significant DVR value of 1.18 for transgenic mice brain compared with that of 1.03 for WT brains. These smaller differences in DVR values of targeted regions in transgenic mice compared with that of WT counterparts could also be attributed to a lack of true negative reference region in aged PS1/APP mice^57^. Similar to [^11^C]-PiB^58^,^59^ and [^18^F]-Florbetaben^60^, [^18^F]-**9** shows also considerable retention in extracerebral regions, such as nasal and eye cavities, consistent with presence of plaques in these rodent models. While literature precedents indicate that [^11^C]-PiB^61^ and [^18^F]-Flutemetamol^62^ did not show differences consistent with spatial localization of Aβ in brains of PS1/APP mice models compared with controls, [^18^F]-**9** shows statistically significant differences between PS1/APP transgenic mice and age-matched WT brains. The differences between these tracers in this rodent model could be attributed to ability of [^18^F]-**9** for targeting both diffuse and fibrillary plaques. To further evaluate, whether or not [^18^F]-**9** offers significant advantage over other approved tracers, the agent would need to be further investigated for its performance in other AD rodent models (APP23 and Tg2576). Nevertheless, the preclinical data indicate that [^18^F]-**9** could provide a novel benzoselenazole based PET tracer, worthy of further evaluation to enable interrogation of Aβ load within the brain.

## Conclusions

A novel heterocyclic benzoselenazole derivative **5** was synthesized and structurally characterized. Crystal structure revealed a planar geometry^25^ with fluorine atoms deviating out of the plane, and the presence of intermolecular and intramolecular hydrogen bonding. The agent demonstrates potent binding affinity to autopsy confirmed AD homogenates and the binding affinity constant is superior to that of [^18^F]-AV-45^41^, [^18^F]-Florbetaben^40^, and [^18^F]-Flutemetamol^42^. Competitive binding displacement experiments indicate that [^18^F]-**9** tracer targets the high affinity binding site on AD homogenates, visualized by [^11^C]-PiB via PET imaging (data not shown). Importantly, [^18^F]-**9** labels Aβ plaques in cortex of autopsy confirmed AD brain sections, while also demonstrating a close immunohistochemical correlation with anti Aβ antibody-conjugate thereby indicating specificity for mapping the biomarker protein. The fluorescent molecule **5** detects Aβ plaques in brain hippocampus sections of transgenic mice, while also visualizing CAA. Noticeably, **5** labels both diffuse and fibrillar plaques in brain sections of AD patients, while lacking interaction with biomarker proteins of other neurodegenerative diseases thereby indicating specificity for detecting Aβ in AD. The ability of **5** to detect diffuse plaques (the precursor to fibrillar neuritic plaques) in AD brain tissue sections could be beneficial in stratification of subjects with a risk factor for development of AD, while also enabling quantitative assessment of overall efficacy of anti-amyloid therapeutics. Finally, Fluselenamyl demonstrates favorable biological half-life in human serum, facile brain penetration, and ability to detect parenchymal plaques in transgenic mice, minutes post-intravenous injection. Overall, these data provide a provocative platform for further development of PET tracers comprising benzoselenazoles moiety in their organic scaffold to enable noninvasive assessment of Aβ plaques *in vivo*. Further investigations evaluating ability of **5** to map Aβ burden in various AD rodent models as a function of aging are under progress.

## Additional Information

**How to cite this article**: Sundaram, G. S. M. *et al.* Fluselenamyl: A Novel Benzoselenazole Derivative for PET Detection of Amyloid Plaques (Aβ) in Alzheimer’s Disease. *Sci. Rep.* **6**, 35636; doi: 10.1038/srep35636 (2016).

**Publisher’s note:** Springer Nature remains neutral with regard to jurisdictional claims in published maps and institutional affiliations.

## Supplementary Material

Supplementary Information

## References

[b1] Ferri C. P. *et al.* Global prevalence of dementia: a Delphi consensus study. Lancet 366, 2112–2117 (2005). 16360788 10.1016/S0140-6736(05)67889-0 PMC2850264

[b2] Cummings J. L. Biomarkers in Alzheimer’s disease drug development. Alzheimer’s & dementia: the journal of the Alzheimer’s Association 7, e13–e44, doi: 10.1016/j.jalz.2010.06.004 (2011). 21550318

[b3] Teipel S. J., Buchert R., Thome J., Hampel H. & Pahnke J. Development of Alzheimer-disease neuroimaging-biomarkers using mouse models with amyloid-precursor protein-transgene expression. Prog. Neurobiol. 95, 547–556, doi: 10.1016/j.pneurobio.2011.05.004 (2011). 21601614

[b4] Prvulovic D. & Hampel H. Amyloid beta (Abeta) and phospho-tau (p-tau) as diagnostic biomarkers in Alzheimer’s disease. Clin. Chem. Lab. Med. 49, 367–374, doi: 10.1515/CCLM.2011.087 (2011). 21342022

[b5] Hampel H. *et al.* Biomarkers for Alzheimer’s disease therapeutic trials. Prog. Neurobiol. 95, 579–593, doi: 10.1016/j.pneurobio.2010.11.005 (2011). 21130138

[b6] Price J. L. *et al.* Neuropathology of nondemented aging: presumptive evidence for preclinical Alzheimer disease. Neurobiol. Aging 30, 1026–1036, doi: 10.1016/j.neurobiolaging.2009.04.002 (2009). 19376612 PMC2737680

[b7] Klunk W. *et al.* Imaging brain amyloid in Alzheimer’s disease with Pittsburgh Compound-B. Ann. Neurol 55, 306–319 (2004). 14991808 10.1002/ana.20009

[b8] Shin J., Kepe V., Barrio J. R. & Small G. W. The merits of FDDNP-PET imaging in Alzheimer’s disease. Journal of Alzheimer’s disease: JAD 26 Suppl 3, 135–145, doi: 10.3233/JAD-2011-0008 (2011). 21971458

[b9] Verhoeff N. *et al.* *In vivo* imaging of Alzheimer disease β-amyloid with [^13^C]SB-13 PET. Am. J. Geriatr. Psychiatry 12, 584–595 (2004). 15545326 10.1176/appi.ajgp.12.6.584

[b10] Choi S. R. *et al.* Preclinical properties of 18F-AV-45: a PET agent for Abeta plaques in the brain. J. Nucl. Med. 50, 1887–1894, doi: 10.2967/jnumed.109.065284 (2009). 19837759 PMC3065020

[b11] Rowe C. C. *et al.* Head-to-head comparison of 11C-PiB and 18F-AZD4694 (NAV4694) for beta-amyloid imaging in aging and dementia. J. Nucl. Med. 54, 880–886, doi: 10.2967/jnumed.112.114785 (2013). 23575995

[b12] Cselenyi Z. *et al.* Clinical validation of 18F-AZD4694, an amyloid-beta-specific PET radioligand. J. Nucl. Med. 53, 415–424, doi: 10.2967/jnumed.111.094029 (2012). 22323782

[b13] Nordberg A. PET imaging of amyloid in Alzheimer’s disease. Lancet neurology 3, 519–527 (2004). 15324720 10.1016/S1474-4422(04)00853-1

[b14] Maya Y. *et al.* Preclinical properties and human *in vivo* assessment of 123I-ABC577 as a novel SPECT agent for imaging amyloid-beta. Brain 139, 193–203, doi: 10.1093/brain/awv305 (2016). 26490333 PMC4949387

[b15] Chen C. J. *et al.* *In vivo* SPECT imaging of amyloid-beta deposition with radioiodinated imidazo[1,2-a]pyridine derivative DRM106 in a mouse model of Alzheimer’s disease. J. Nucl. Med. 56, 120–126, doi: 10.2967/jnumed.114.146944 (2015). 25476539

[b16] Hsiao I. T. *et al.* Correlation of early-phase 18F-florbetapir (AV-45/Amyvid) PET images to FDG images: preliminary studies. Eur J Nucl Med Mol Imaging 39, 613–620, doi: 10.1007/s00259-011-2051-2 (2012). 22270508

[b17] Koole M. *et al.* Whole-body biodistribution and radiation dosimetry of 18F-GE067: a radioligand for *in vivo* brain amyloid imaging. J. Nucl. Med. 50, 818–822, doi: 10.2967/jnumed.108.060756 (2009). 19372469

[b18] Nelissen N. *et al.* Phase 1 study of the Pittsburgh compound B derivative 18F-flutemetamol in healthy volunteers and patients with probable Alzheimer disease. J. Nucl. Med. 50, 1251–1259, doi: 10.2967/jnumed.109.063305 (2009). 19617318

[b19] Rowe C. C. *et al.* Imaging of amyloid beta in Alzheimer’s disease with 18F-BAY94-9172, a novel PET tracer: proof of mechanism. Lancet neurology 7, 129–135, doi: 10.1016/S1474-4422(08)70001-2 (2008). 18191617

[b20] Villemagne V. L. *et al.* Amyloid imaging with (18)F-florbetaben in Alzheimer disease and other dementias. J. Nucl. Med. 52, 1210–1217, doi: 10.2967/jnumed.111.089730 (2011). 21764791

[b21] Becker G. A. *et al.* PET quantification of 18F-florbetaben binding to beta-amyloid deposits in human brains. J. Nucl. Med. 54, 723–731, doi: 10.2967/jnumed.112.107185 (2013). 23471310

[b22] Wong D. F. *et al.* *In vivo* imaging of amyloid deposition in Alzheimer disease using the radioligand 18F-AV-45 (florbetapir [corrected] F 18). J. Nucl. Med. 51, 913–920, doi: 10.2967/jnumed.109.069088 (2010). 20501908 PMC3101877

[b23] Ni R., Gillberg P. G., Bergfors A., Marutle A. & Nordberg A. Amyloid tracers detect multiple binding sites in Alzheimer’s disease brain tissue. Brain 136, 2217–2227, doi: 10.1093/brain/awt142 (2013). 23757761

[b24] Lockhart A. *et al.* Evidence for presence of three distinct binding sites for thioflavin T class of Alzheimer’s disease PET imaging agents on β-amyloid peptide fibrils. J. Biol. Chem. 280, 7677–7684 (2005). 15615711 10.1074/jbc.M412056200

[b25] Sundaram G. *et al.* Characterization of a brain permeant fluorescent molecule and visualization of Aβ parenchymal plaques, using real-time multiphoton imaging in transgenic mice. Org. Lett. 16, 3640–3643 (2014). 25003699 10.1021/ol501264q PMC4372081

[b26] Sundaram G., Cairns N., Lee J.-M. & Sharma V. Design and synthesis of a novel PET probe for early detection of Alzheimer’s disease. J. Nucl. Med. 55, 137 (2014).

[b27] Sundaram G. S. *et al.* Synthesis, characterization, and preclinical validation of a PET radiopharmaceutical for interrogating Abeta (beta-amyloid) plaques in Alzheimer’s disease. EJNMMI research 5, 112, doi: 10.1186/s13550-015-0112-4 (2015). 26061601 PMC4478171

[b28] Fujiwara S., Asanuma Y., Shin-ike T. & Kambe N. Copper(I)-catalyzed highly efficient synthesis of benzoselenazoles and benzotellurazoles. J. Org. Chem. 72, 8087–8090, doi: 10.1021/jo7013164 (2007). 17867702

[b29] Redon S., Kabri Y., Crozet M. & Vanelle P. One pot preparation of 2-(alkyl)arylbenzoselenazoles from the coressponding N-(acetyl)benzoyl-2-iodoanilines via a microwave assisted methodology. Tet Lett 55, 5052–5054 (2014).

[b30] Yu K., Park J. & Yang S. Synthesis of [^18^F]Fluorocholine analogues as potential imaging agents for PET studies. Bull Korean Chem Soc 25, 506–510 (2004).

[b31] Carrera I. *et al.* Vaccine Development to Treat Alzheimer’s Disease Neuropathology in APP/PS1 Transgenic Mice. International journal of Alzheimer’s disease 2012, 376138, doi: 10.1155/2012/376138 (2012). PMC3457670 23024882

[b32] Tanifum E. A. *et al.* Intravenous delivery of targeted liposomes to amyloid-beta pathology in APP/PSEN1 transgenic mice. PloS one 7, e48515, doi: 10.1371/journal.pone.0048515 (2012). 23119043 PMC3485335

[b33] DeMattos R., O’dell M., Parsadanian M., Holtzman D. *et al.* Clusterin promotes amyloid plaque formation and is critical for neuritic toxicity in a mouse model of Alzheimer’s disease. Proc Natl Acad Sci USA 99, 10843–10848 (2002). 12145324 10.1073/pnas.162228299 PMC125060

[b34] Cirrito J. R. *et al.* Serotonin signaling is associated with lower amyloid-beta levels and plaques in transgenic mice and humans. Proc. Natl. Acad. Sci. USA 108, 14968–14973, doi: 10.1073/pnas.1107411108 (2011). 21873225 PMC3169155

[b35] Mirra S. *et al.* The Consortium to Establish a Registry for Alzheimer’s Disease (CERAD). Part II. Standardization of the neuropathologic assessment of Alzheimer’s disease. Neurology 41, 479–486 (1991). 2011243 10.1212/wnl.41.4.479

[b36] Hyman B. & Trojanowski J. Consensus recommendations for the postmortem diagnosis of Alzheimer disease from the National Institute on Aging and the Reagan Institute Working Group on diagnostic criteria for the neuropathological assessment of Alzheimer disease. J. Neuropathol. Exp. Neurol. 56, 1095–1097 (1997). 9329452 10.1097/00005072-199710000-00002

[b37] Cairns N. J., Taylor-Reinwald L. & Morris J. C. Autopsy consent, brain collection, and standardized neuropathologic assessment of ADNI participants: the essential role of the neuropathology core. Alzheimer’s & dementia: the journal of the Alzheimer’s Association 6, 274–279, doi: 10.1016/j.jalz.2010.03.012 (2010). PMC2893399 20451876

[b38] Mathis C. A., Mason N. S., Lopresti B. J. & Klunk W. E. Development of positron emission tomography beta-amyloid plaque imaging agents. Semin. Nucl. Med. 42, 423–432, doi: 10.1053/j.semnuclmed.2012.07.001 (2012). 23026364 PMC3520098

[b39] Bagchi D. P. *et al.* Binding of the radioligand SIL23 to alpha-synuclein fibrils in Parkinson disease brain tissue establishes feasibility and screening approaches for developing a Parkinson disease imaging agent. PloS one 8, e55031, doi: 10.1371/journal.pone.0055031 (2013). 23405108 PMC3566091

[b40] Highlights of prescribing information for Neuraceq (Florbetaben). http://www.accessdata.fda.gov/drugsatfda_docs/label/2014/204677s000lbl.pdf (2014).

[b41] Choi S. R. *et al.* Correlation of amyloid PET ligand florbetapir F 18 binding with Abeta aggregation and neuritic plaque deposition in postmortem brain tissue. Alzheimer Dis. Assoc. Disord. 26, 8–16, doi: 10.1097/WAD.0b013e31821300bc (2012). 22354138 PMC3286131

[b42] Brown P. Pharmacology/toxicology NDA review and evaluation for Vizamyl (Flutemetamol). http://www.accessdata.fda.gov/drugsatfda_docs/nda/2013/203137Orig1s000PharmR.pdf (2013).

[b43] Johnson A. E. *et al.* AZD2184: a radioligand for sensitive detection of beta-amyloid deposits. J. Neurochem. 108, 1177–1186, doi: 10.1111/j.1471-4159.2008.05861.x (2009). 19141073

[b44] Matsumura K. *et al.* Structure-Activity Relationship Study of Heterocyclic Phenylethenyl and Pyridinylethenyl Derivatives as Tau-Imaging Agents That Selectively Detect Neurofibrillary Tangles in Alzheimer’s Disease Brains. J. Med. Chem. 58, 7241–7257, doi: 10.1021/acs.jmedchem.5b00440 (2015). 26327138

[b45] Mathis C. *et al.* Synthesis and evaluation of ^11^C-labeled 6-substituted 2-arylbenzothiazoles as amyloid imaging agents. J. Med. Chem. 46, 2740–2754 (2003). 12801237 10.1021/jm030026b

[b46] Brockschnieder D. *et al.* Preclinical characterization of a novel class of 18F-labeled PET tracers for amyloid-beta. J. Nucl. Med. 53, 1794–1801, doi: 10.2967/jnumed.112.104810 (2012). 23008501

[b47] Dischino D., Welch M., Kilbourn M. & Raichle M. Relationship between lipophilicity and brain extraction of C-11 labeled radiopharmaceuticals. J Nucl Med 24, 1030–1038 (1983). 6605416

[b48] Quillard T. & Libby P. Molecular imaging of atherosclerosis for improving diagnostic and therapeutic development. Circ. Res. 111, 231–244, doi: 10.1161/CIRCRESAHA.112.268144 (2012). 22773426 PMC3412367

[b49] Dong J., Revilla-Sanchez R., Moss S. & Haydon P. G. Multiphoton *in vivo* imaging of amyloid in animal models of Alzheimer’s disease. Neuropharmacology 59, 268–275, doi: 10.1016/j.neuropharm.2010.04.007 (2010). 20398680 PMC3117428

[b50] Janus C., Chishti M. A. & Westaway D. Transgenic mouse models of Alzheimer’s disease. Biochim. Biophys. Acta 1502, 63–75 (2000). 10899432 10.1016/s0925-4439(00)00033-8

[b51] F v. L. Single and multiple transgenic mice as models for Alzheimer’s disease. Prog. Neurobiol 61, 305–312 (2000). 10727777 10.1016/s0301-0082(99)00055-6

[b52] Holtzman D., Bales K., Tenkova T., Paul S. *et al.* Apolipoprotein E isoform-dependent amyloid deposition and neuritic degeneration in a mouse model of Alzheimer’s disease. Proc Natl Acad Sci USA 97, 2892–2897 (2000). 10694577 10.1073/pnas.050004797 PMC16026

[b53] Gotz J. Tau and transgenic animal models. Brain Res. Brain Res. Rev. 35, 266–286 (2001). 11423157 10.1016/s0165-0173(01)00055-8

[b54] Crews L., Rockenstein E. & Masliah E. APP transgenic modeling of Alzheimer’s disease: mechanisms of neurodegeneration and aberrant neurogenesis. Brain structure & function 214, 111–126, doi: 10.1007/s00429-009-0232-6 (2010). 20091183 PMC2847155

[b55] Kurt M. A. *et al.* Neurodegenerative changes associated with beta-amyloid deposition in the brains of mice carrying mutant amyloid precursor protein and mutant presenilin-1 transgenes. Exp. Neurol. 171, 59–71, doi: 10.1006/exnr.2001.7717 (2001). 11520121

[b56] Bacskai B. *et al.* Four-dimensional multiphoton imaging of brain entry, amyloid binding and clearance of an amyloid-β ligand in transgenic mice. Proc Natl Acad Sci USA 100, 12462–12467 (2003). 14517353 10.1073/pnas.2034101100 PMC218780

[b57] Brendel M. *et al.* Cross-sectional comparison of small animal [18F]-florbetaben amyloid-PET between transgenic AD mouse models. PloS one 10, e0116678, doi: 10.1371/journal.pone.0116678 (2015). 25706990 PMC4338066

[b58] Maeda J. *et al.* Longitudinal, quantitative assessment of amyloid, neuroinflammation, and anti-amyloid treatment in a living mouse model of Alzheimer’s disease enabled by positron emission tomography. J. Neurosci. 27, 10957–10968, doi: 10.1523/JNEUROSCI.0673-07.2007 (2007). 17928437 PMC6672864

[b59] Manook A. *et al.* Small-animal PET imaging of amyloid-beta plaques with [11C]PiB and its multi-modal validation in an APP/PS1 mouse model of Alzheimer’s disease. PloS one 7, e31310, doi: 10.1371/journal.pone.0031310 (2012). 22427802 PMC3302888

[b60] Rominger A. *et al.* Longitudinal assessment of cerebral beta-amyloid deposition in mice overexpressing Swedish mutant beta-amyloid precursor protein using 18F-florbetaben PET. J. Nucl. Med. 54, 1127–1134, doi: 10.2967/jnumed.112.114660 (2013). 23729696

[b61] Snellman A. *et al.* Longitudinal amyloid imaging in mouse brain with 11C-PIB: comparison of APP23, Tg2576, and APPswe-PS1dE9 mouse models of Alzheimer disease. J. Nucl. Med. 54, 1434–1441, doi: 10.2967/jnumed.112.110163 (2013). 23833271

[b62] Snellman A. *et al.* *In vivo* PET imaging of beta-amyloid deposition in mouse models of Alzheimer’s disease with a high specific activity PET imaging agent [(18)F]flutemetamol. EJNMMI research 4, 37, doi: 10.1186/s13550-014-0037-3 (2014). 25977876 PMC4412375

