# Supplementary material for: Fluselenamyl: A Novel Benzoselenazole Derivative for PET Detection of Amyloid Plaques (Aβ) in Alzheimer’s Disease
Source: Sci Rep. 2016 Nov 2;6:35636. doi: 10.1038/srep35636 (PMC5090206; doi:10.1038/srep35636)
Supplement: Supplementary Information [file srep35636-s1.pdf]

Supporting Information (Sundaram et. al.)

Fluselenamyl: A Novel Benzoselenazole Derivative for PET Detection of Amyloid Plaques (Aβ) in Alzheimer's Disease

GSM Sundaram[1,2]

Dhruva Dhavale[3,4]

Julie L. Prior[1,2]

Ping Yan[3,4]

John Cirrito[3,4,5]

Nigam P. Rath[6]

Richard Laforest[2]

Nigel J. Cairns[5,7]

Jin-Moo Lee[2,3,4,8]

Paul T. Kotzbauer[3,4]

Vijay Sharma[1,2,3,8]*

[1]ICCE Institute, Molecular Imaging Center, Washington University School of Medicine, MO 63110, USA
[2]Mallinckrodt Institute of Radiology, Washington University School of Medicine, MO 63110, USA
[3]Department of Neurology, Washington University School of Medicine, MO 63110, USA
[4]Hope Center for Neurological Disorders, Washington University School of Medicine, MO 63110, USA
[5]Knight Alzheimer's Disease Research Center, Washington University School of Medicine, MO 63110, USA
[6]Departments of Chemistry & Biochemistry, University of Missouri, St. Louis, MO 63121, USA
[7]Departments of Pathology & Immunology, Washington University School of Medicine, MO 63110, USA
[8]Department of Biomedical Engineering, School of Engineering & Applied Science, Washington University, St. Louis 63105, USA.

*Correspondence: Vijay Sharma, Ph.D.
Mallinckrodt Institute of Radiology
Washington University School of Medicine, Box 8225
510 S. Kingshighway Blvd.
St. Louis, MO 63110
Tele: 314-362-9358, Fax: 314-362-0152
Email: sharmav@mir.wustl.edu

Experimental Section

General Methods.

All reagents were purchased from Sigma-Aldrich unless otherwise stated. 2-fluoroethyl-4-methylbenzene sulfonate was prepared using literature procedure. [1] ^{1}H NMR, proton-decoupled ^{13}C NMR, and ^{19}F NMR spectra were recorded on a Varian 400 MHz spectrometer; chemical shifts are reported in δ (ppm) with reference to either TMS or trichlorofluoromethane ($CFCl_3$). Mass spectra were obtained from the University of Missouri, Mass Spectrometry facility using nitrobenzyl alcohol (NBA) as matrix and analyzed via HRFab. Purity of the **5** was assessed using an HPLC (Waters system 600 equipped with dual λ-detector 2487 set to 254 and 280 nm) with a C-18 reversed-phase column (Phenomenex Luna®- C18; 100 Å; 5 µm; 250 x 10 mm) using an eluent mixture of acetonitrile and water as a gradient system (75% acetonitrile in water over 20min) at a flow of 3 mL/min). For characterization of ^{18}F-**9**, HPLC flow detector was also interfaced with a radio-detector (BioScans) for analysis of radiolabeled chemical entities.

Chemical Synthesis.

5-methoxy-2-methylbenzo[d][1,3]selenazole (2).

2 was obtained from N-(acetyl)benzoyl-2-iodoaniline using Woolins reagent under microwave conditions using established procedures. [2]

(Z)-4-(2-(5-methoxybenzo[d][1,3]selenazol-2-yl)vinyl)-N,N-dimethylaniline (3)

To a mixture of 5-methoxy-2-methyl benzoselenazole (0.23 g, 1.0 mmol) and 6-dimethylamino pyrimidine carbaldehyde (0.15 g, 1.0 mmol) in DMSO was added 50 % KOH aqueous solution (10 mL). The resulting mixture was stirred at room temperature for 12 h. After the completion of the reaction, the reaction mixture was filtered and the resulted solid was washed with water (3 × 5mL). The wet solid was re-dissolved in DCM (15 mL), washed with water (3 × 5 mL), and combined organic extract was dried over the sodium sulfate. The contents were filtered and concentrated under reduced pressure to obtain a yellow solid (**2;** 0.33 g; 94%; R*f* = 0.60 , 3:2 hexane: EtOAc) and used for the next step without purification.

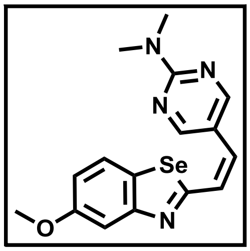

^{1}H NMR (400 MHz, $CDCl_3$): δ 3.24 (s, 6H), 3.87 (s, 3H), 6.93 (d, *J* = 7.2 Hz, 1H), 7.16 (s, 2H), 7.50 (s, 1H), 7.70 (d, *J* = 7.4 Hz, 1H), 8.52 (s, 2H); ^{13}C NMR (100 MHz, $CDCl_3$): 37.27, 55.52, 107.18, 114.85, 117.07, 121.54, 124.81, 128.10, 132.81, 156.54, 159.16, 161.64, 172.49; HRMS (FAB) m/z calc. for $C_{16}H_{17}N_4OSe$: $[M+1]^+$ 361.0567; found: 361.0572.

(Z)-2-(4-(dimethylamino)styryl)benzo[d][1,3]selenazol-5-ol (4)

The condensed product **3** (0.1 g, 0.3 mmol) was dissolved in dry DCM (5mL) under argon, cooled to -78 °C, stirred for 5 min, and treated with drop-wise addition of BBr_3 (1M in DCM, 0.15 mL, 5.0 mmol). The resulting mixture was slowly brought to room temperature and stirred overnight. Following completion of the reaction (monitored by TLC), the reaction mixture was cooled to 0 °C and quenched with cold satd. sodium bicarbonate solution (5 mL). The reaction mixture was extracted with ethyl acetate (2 × 25 mL), combined extract was washed with water (2 × 10 mL), dried over anhydrous sodium sulfate, filtered, and the filtrates were evaporated under reduced pressure. The residual red solid was purified by flash chromatography using hexane: EtOAc : MeOH (10: 9: 1) as an eluent mixture to obtain **4** (0.09 g; 90%; R*f* = 0.21)

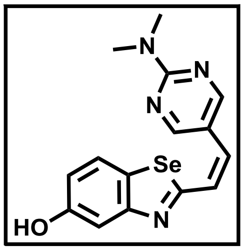

^{1}H NMR (400 MHz, DMSO-d_6): δ 3.17 (s, 6H), 6.83 (dd, *J* = 8.0, 1.6 Hz, 1H), 7.30 (s, 1H) 7.40 (d, *J* = 8.8 Hz, 2H), 7.82 (d, *J* = 8.8 Hz, 1H), 8.75 (s, 2H), 9.63 (s, 1H); ^{13}C NMR (100 MHz, DMSO-d_6): 39.28, 109.72, 115.23,

117.66, 121.91, 125.92, 126.55, 133.49, 156.95, 157.07, 157.49, 161.59, 172.49; HRMS (FAB) m/z calc. for $C_{15}H_{15}N_4OSe$: $[M+1]^+$ 347.0411; found: 347.0412.

(E)-5-(2-(6-(2-fluoroethoxy)benzo[d]thiazol-2-yl)vinyl)-N,N-dimethylpyridin-2-amine (**5**)

To a solution of alcohol derivative **4** (0.17 g, 0.5 mmol) in DMF (2mL) and 2-fluoroethyltosylate [1] (0.1g, 0.5 mmol) dissolved in DMF (3mL) was added Cs_2CO_3 (0.24 g, 0.75 mmol). The contents were stirred at 130°C for 5h. Following completion of the reaction (monitored by TLC), the reaction mixture was quenched with ice-cold water and extracted with ethyl acetate (3 ×25 mL). The combined organic layer was washed with water (2 × 50 mL), dried over anhydrous sodium sulfate, filtered, and the filtrate evaporated under reduced pressure. The crude residue was purified by PTLC using hexane : EtOAc as an eluent mixture (60 : 40) to obtain **5** (0.13 g; 67% ; bright yellow solid; R*f* = 0.41; 3:2, EtOAc-hexane).

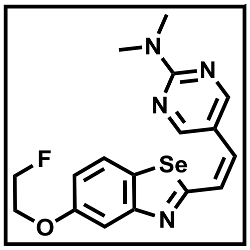

^{1}H NMR (400 MHz, $CDCl_3$): 3.24 (s, 6H), 4.28 (d, *J* = 27.6 Hz, 2H), 4.80 (d, *J* = 47.2 Hz, 2H), 6.97 (d, *J* = 7.2 Hz, 1H), 7.17 (bs, 2H), 7.50 (s, 1H), 7.72 (d, *J* = 7.2 Hz, 1H), 8.52 (s, 2H); ^{13}C NMR (100 MHz, $CDCl_3$): 37.28, 67.31, 67.51, 80.97, 82.67, 107.95, 115.36, 117.04, 121.46, 125.01, 128.78, 133.01, 156.57, 157.93, 158.57, 161.66, 172.72; ^{19}F NMR (282 MHz, $CFCl_3$): -224 ppm; HRMS (FAB) m/z calc. for $C_{17}H_{18}FN_4OSe$: $[M]^+$ 393.0630; found: 393.0622.

(Z)-5-(2-(5-(2-((tert-butyldimethylsilyl)oxy)ethoxy)benzo[d][1,3]selenazol-2-yl)vinyl)-N,N-dimethylpyrimidin-2-amine (**6**)

To the solution of alcohol **3** (0.14 g, 0.4 mmol) and 2-(Bromoethoxy)-tert-butyldimethylsilane (0.096g, 0.4 mmol) in DMF (5mL) was added Cs_2CO_3 (0.20 g, 0.6 mmol). The resulting mixture was stirred at 140 °C for 6h. Following the completion of the reaction (monitored by TLC), it was quenched with the addition of ice cold water and extracted with ethyl acetate (3 X 25 mL). The organic layer was washed with water (2 X 50 mL) and dried over anhydrous sodium sulfate and the solvent evaporated under reduced pressure to give crude product which were purified by PTLC using hexane : EtOAc (60 : 40) as eluent; Yield 61% (0.12 g); Straw Yellow Solid ; R*f* 0.45 (2:3 hexane-EtOAc);

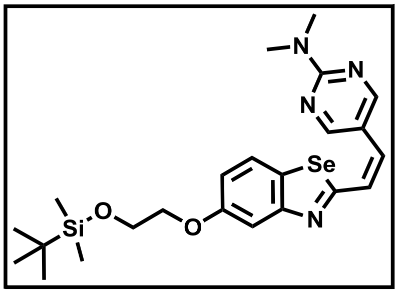

^{1}H NMR (400 MHz, $CDCl_3$): 0.12 (s, 6H), 0.92 (s, 9H), 3.24 (s, 6H), 4.01-4.03 (m, 2H), 4.09-4.12 (m, 2H), 6.94 (dd, *J* = 8.8, 2.4 Hz, 1H), 7.17 (d, *J* = 1.2 Hz, 2H), 7.50 (d, *J* = 2.4 Hz, 1H), 7.70 (d, *J* = 8.4 Hz, 1H), 8.53 (s, 2H) ; ^{13}C NMR (100 MHz, $CDCl_3$): 31.04, 42.24, 68.44, 76.22, 110.76, 112.22, 121.21, 124.34, 129.86, 140.02, 142.74, 149.96, 152.44, 162.68, 172.86; HRMS (FAB) m/z calc. for $C_{23}H_{33}N_4O_2SiSe$: $[M]^+$ 505.1538; found: 505.1527.

(Z)-2-((2-(4-(dimethylamino)styryl)benzo[d][1,3]selenazol-5-yl)oxy)ethan-1-ol (**7**)

To the solution of TBDMS protected compound (0.05 g, 0.1 mmol) in THF was added TBAF (1M in THF, 0.5 mL, 0.5 mmol) and stirred at RT for 6h. Once the reaction is completed (monitored by TLC), the solvent is evaporated under reduced pressure to give crude product which was purified by PTLC using EtOAC: Hex (75:25) as eluent; Yield 81% (0.032 g); Dark Yellow Solid; R*f* 0.32 (2:3 hexane-EtOAc);

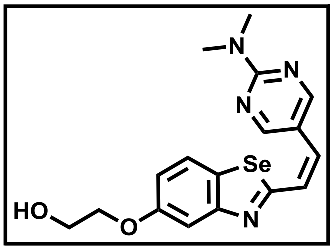

^{1}H NMR (400 MHz, $CDCl_3$): 3.24 (s, 6H), 3.99-4.01 (m, 2H), 4.14-4.16 (m, 2H), 6.68 (d, *J* = 12.0, Hz, 1H), 6.75 (d, *J* = 12.0, Hz, 1H), 6.94 (dd, *J* = 8.8, 2.4 Hz, 1H), 7.56 (d, *J* = 2.8 Hz, 1H), 7.65 (d, *J* = 9.2 Hz, 1H), 8.65 (s, 2H) ^{13}C NMR (100 MHz, $CDCl_3$): 37.24, 61.35, 69.61, 108.29, 115.57, 117.06, 121.49, 124.94, 125.45, 130.94, 132.99, 155.85, 156.57, 158.05, 158.55, 169.12, 172.72; HRMS (FAB) m/z calc. for $C_{17}H_{19}N_4O_2Se$: $[M]^+$ 391.0673; found: 391.0682.

(Z)-2-((2-(2-(2-(dimethylamino)pyrimidin-5-yl)vinyl)benzo[d][1,3]selenazol-5-yl)oxy)ethyl 4-methylbenzenesulfonate (8)

Pyridine (0.08 g, 1 mmol) and DMAP (0.0012 g, 0.01 mmol) were added to a solution of alcohol **6** (0.08g, 0.2 mmol) in DCM (10 mL) at 0°C. Thereafter, *p*-toluene-sulfonylchloride (0.076 g, 0.4 mmol) dissolved in DCM (2 mL) was added and the resulting solution was stirred at room temperature for 7 h, and quenched by the addition of water (15 mL). The resulting mixture was extracted with DCM (3 X 5 mL), organic extracts were combined, dried over Na_2SO_4, filtered, and concentrated. Finally, the residue was purified by Prep TLC using the eluent mixture (Hex/EtOAc = 60 : 40) to obtain the compound as a Dark yellow solid. Yield 63% (0.07 g); Dark yellow solid; R*f* 0.39 (1:1 hexane-EtOAc):

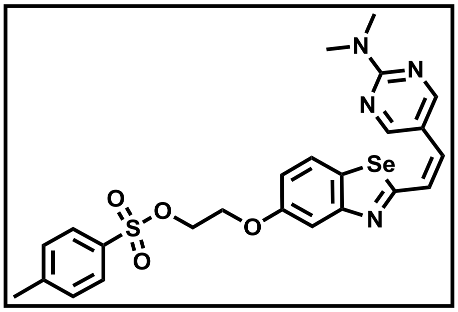

^{1}H NMR (400 MHz, $CDCl_3$): 2.43 (s, 3H), 3.24 (s, 6H), 4.20 (bs, 2H), 4.41 (bs, 2H), 6.71 (d, *J* = 9.0 Hz, 1H), 6.81 (d, *J* = 9.0 Hz, 1H), 7.17-7.41 (m, 4H), 7.65-7.69 (m, 1H), 7.82 (d, *J* = 7.6 Hz, 2H),8.52 (s, 1H), 8.66 (s, 1H) ; ^{13}C NMR (100 MHz, $CDCl_3$): 21.64, 37.23, 65.72, 68.06, 108.04, 108.25, 115.08, 115.50, 116.88, 121.41, 124.78, 124.96, 125.25, 128.00, 129.85, 131.04, 133.12, 144.95, 156.60, 157.35, 158.59, 162.68, 171.84; ; HRMS (FAB) m/z calc. for $C_{24}H_{24}FN_4O_4SNaSe$: $[M]^+$ + Na 567.0581; found: 567.0580.

(E)-5-(2-(6-(2-fluoroethoxy)benzo[d]thiazol-2-yl)vinyl)-N,N-dimethylpyridin-2-amine (**5**)

Dry tetrabutylammonium fluoride (0.065g, 0.25 mmol) was dissolved in dry acetonitrile (5 mL) under argon and treated with the acetonitrile solution of tosylated precursor **8** (0.054g, 0.1 mmol) and refluxed at 110 °C. The progress of the reaction was monitored via the TLC. Following completion of the reaction, the solvent was evaporated and the residue was extracted with EtOAc (2 X 10 mL). Combined organic extracts were dried with Na_2SO_4, filtered, evaporated, and the residue was purified using thinlayer chromatography employing a mobile eluent mixture (Hex : EtOAC = 60:40) to obtain **4** (0.028 g; 71% ; bright yellow solid; R*f* = 0.41; 3:2, EtOAc-hexane).

X-ray Crystallography.

X-ray quality Crystals of **5** were obtained by slow diffusion of MeOH to a DCM solution of **5** containing 1% ACN. A crystal of approximate dimensions 0.594 x 0.076 x 0.054 mm^3 was mounted on a MiTeGen cryoloop in a random orientation. Preliminary examination and data collection were performed using a Bruker X8 Kappa Apex II Charge Coupled Device (CCD) Detector system single crystal X-Ray diffractometer, equipped with an Oxford Cryostream LT device. Data were collected using graphite monochromated Mo K α radiation (λ= 0.71073 Å) from a fine focus sealed tube X-Ray source. Preliminary unit cell constants were determined with a set of 36 narrow frame scans. Typical data sets consist of combinations of ϖ and ϕ scan frames with typical scan width of 0.5° and counting time of 15 seconds/frame at a crystal to detector

distance of 4.0 cm. The collected frames were integrated using an orientation matrix determined from the narrow frame scans. Apex II and SAINT software packages [3] were used for data collection and data integration. Analysis of the integrated data did not show any decay. Final cell constants were determined by global refinement of reflections harvested from the complete data set. Collected data were corrected for systematic errors using SADABS and TWINABS [3] based on the Laue symmetry using equivalent reflections.

Crystal data and intensity data collection parameters are listed in **Table 1**.

Structure solution and refinement were carried out using the SHELXTL- PLUS software package. [4] The structure was solved by direct methods and refined successfully in the space groups, $P2_1/n$. Twin refinement was carried out for a two component twin. Full matrix least-squares refinements were carried out by minimizing $\Sigma w(F_o^2-F_c^2)^2$. The non-hydrogen atoms were refined anisotropically to convergence. All hydrogen atoms were treated using appropriate riding model (AFIX m3). The final residual values (R1= 0.063, wR2= 0.171) and structure refinement parameters are listed in **Table 1**. Furthermore, a molecule of CH_3CN was also located in the lattice and refined with partial occupancies and geometrical restraints. The crystal structure of **5** revealed a planar geometry with fluoride atom displaced from the mean plane and presence of both inter- and intra-molecular hydrogen bonding **(SI-Figure 1).** Complete listings of structural refinement parameters, atomic coordinates and equivalent isotropic displacement parameters, interatomic distances and bond angles, anisotropic displacement coefficients for the non-hydrogen atoms, hydrogen coordinates and isotropic displacement parameters, and torsion angles for **5** are listed (**Tables 1-6**). Table of calculated and observed structure factors are available in electronic format.

Bioassays.

Preparation of Aβ fibrils.

Commercially available amyloid peptide $A\beta_{1-42}$ (433 μg) was gently dissolved in PBS (1 mL, pH 7.4, 100 μM)[5]. The solution was incubated for 36-48 h at 37°C with continuous gentle shaking to avoid gel formation at the meniscus. The aggregated peptide suspension was stored at -80°C until needed (the suspension did not show any noticeable change in properties for at least 8 weeks). [5]

Binding of 5 with Aβ fibrils

In vitro binding assays to preformed fibrils were performed using literature procedures. [5,6] Prior to binding assays, the Aβ fibril stock solution was thawed, diluted with PBS to the final concentration of Aβ fibrils (1μM), and incubated with increasing concentrations of the fluorescent molecule **5** for 30 min. Following excitation at 410nm, fluorescence spectrum of **5**recorded in PBS containing 5% ethanol showed a broad emission peak 450-540nm with E_{max} at **503**nm. Upon incubation with preformed of $A\beta_{1-42}$ aggregates, the peak at 503nm shifted slightly (E_{max} 485 nm) and showed remarkable enhancement in fluorescence, indicating binding to Aβ aggregates, similar to enhancement in fluorescence of thioflavin-T in PBS (a positive control; data not shown). Additionally, it is also noteworthy that no fluorescence was observed using Aβ aggregates alone in PBS upon excitation at 410 nm (a negative control). All measurements were made in triplicates; fluorescence of either **5** alone or fibrils at a given concentration was subtracted. The data were fit to a single site model using GraphPad Prism, version 4.03 (GraphPad Software, San Diego, California, USA) to obtain the binding constant 1.58±.05nM (**SI-Figure 2**).

Histochemical Staining of Mouse Brain Tissues

Mice, 24 month old $APP^{+/-}/PS1^{+/-}$ and age-matched control BL/6 (WT), were sacrificed and their brains removed, fixed in 4% formaldehyde, and stored in 30% sucrose for 3 days. Serial brain tissue sections (50

µm) were cut in the coronal plane on a freezing sliding microtome and stored at -20°C. [7,8] Tissue was stained as free-floating sections. Prior to staining, tissue sections were permeabilized with PBS-Tritron-X-100 (0.25%) for 30 minutes, and then blocked with 1% non-fat dry milk in PBS-Ttiton-X-100 for 60 minutes. Hippocampus tissue sections of $APP^{+/-}/PS1^{+/-}$ and age-matched control BL/6 (WT, **SI Figure 3)** were incubated with fluorescent molecule **5** (100 nM dissolved in 1% ethanol in PBS) for 60 minutes, and washed 3-times with PBS-Triton-X-100.[9] Thereafter, tissue sections were incubated with mouse-anti-Aβ (mHJ3.4) [7] that was directly conjugated to Alexa 568 for 90 minutes and washed 3-times with PBS-Triton-X-100. Tissue sections were mounted onto SuperFrost Plus slides, dried overnight at room temperature, and then sealed with Fluoromount G (Southern Biotech, Birmingham, AL). Finally, stained sections were imaged using Chroma Filter sets (**5**: FITC Ex 425nm, Em 540nm; anti-Aβ (mHJ3.4) antibody-Alex568: Ex. 560 ± 25nm, Em. 645 ± 35nm) using a Nikon Eclipse E800 epifluorescence microscope.

Histological Staining of Human Brain Tissue

Postmortem brain tissues from autopsy-confirmed AD patients and their approximate age-matched healthy controls were obtained through the Knight Alzheimer's Disease Research Center (ADRC) Washington University School of Medicine and processed according to a protocol approved by institutional ADRC executive committees[10]. All methods were carried out on human tissues in "accordance" with the approved guidelines. Formalin-fixed, paraffin embedded tissue sections were obtained from AD brains and age-matched neurological and neuropathological controls. Brain tissue samples were either fixed in 4% formaldehyde, and stored in 30% sucrose for 3 days and sliced (10 µm) a freezing sliding microtome; or tissue was fixed in 10% neutral buffered formal saline, embedded in paraffin wax, and cut at 10 µm. Tissue sections were incubated with either a fluorescent molecule **5** (10 µM) or treated with anti-Aβ (10D5, dilution: 1:10,000; Eli Lilly, Indianapolis, IN) or thioflavin-S or specific antibodies for biomarker proteins of

other neurodegenerative diseases (**SI, Figure 4A & 4B**). Sections were visualized, processed, and analyzed using a Pascal confocal microscope.

Preparation of Human Brain Homogenates.

Well-established literature procedures were used for preparation of AD homogenates. [11,12] Grey matter was isolated from frozen postmortem frontal cortex tissue by dissection with a scalpel. To prepare insoluble fractions, dissected tissue was sequentially homogenized in four buffers (3 ml/g wet weight of tissue) with glass Dounce tissue grinders (Kimble): 1) High salt (HS) buffer: 50 mM Tris-HCl pH 7.5, 750 mM NaCl, 5 mM EDTA; 2) HS buffer with 1% Triton X-100; 3) HS buffer with 1% Triton X-100 and 1 M sucrose; and 4)50 mM Tris-HCl, pH 7.4. Homogenates were centrifuged at 100,000×g after each homogenization step, the pellet was resuspended, and homogenized in the next buffer in the sequence.

Radiochemical Synthesis ^{18}F-9.

For radiolabeling, tosylated precursor (**7**, 3mg) dissolved in dry acetonitrile (400 µL) was transferred into an amber colored vial containing anhydrous kryptofix 2, 2, 2 (Sigma Chemicals)/K_2CO_3/^{18}F-fluoride, obtained using standard procedures. The reaction mixture was heated to 100°C for 20 min in an oil bath, cooled in ice-cold water, and diluted to 5% acetonitrile in water. The crude mixture was loaded on a C-18 sepPak cartridge (Waters), primed with ethanol (5mL) and water (10mL). The C-18 sep-Pak was washed with water (5mL × 6), 25% acetonitrile (5mL × 4), and finally eluted with 100% acetonitrile (1 mL). The crude mixture was purified on purified using high-performance liquid chromatography (dual λ detection set at 254 and 280 nm) equipped with a radiodetector (Bioscans) using a C-18 column (Phenomenex; 5-µm 250×10 mm) using an eluent gradient acetonitrile 75%-95% over 20 min (flow rate: 3 mL/min). The fraction of ^{18}F-**9** eluting at 11 min (radiochemical purity > 95%; radiochemical yield: 30%; specific activity: 1500-1700 Ci/mmol (**Si Figure 5**); was collected, concentrated, resuspended in ethanol/saline, and employed for bioassays.

Binding Assays.

Binding assays were performed using previously described methods.[11] Briefly, a fixed concentration (0.25 µM/well) of Aβ_{1-42} fibrils was incubated for 1 h at 37 °C with increasing concentrations of [^{18}F]-**9** (0.1 nM-15 nM) in 30 mM Tris-HCl pH 7.4, 0.1% BSA in a reaction volume of 150 µl. A fixed ratio of hot:cold ([^{18}F]-**9** and **5**) was used for all radioligand concentrations. The exact hot:cold ([^{18}F]-**9** : **5**) ratio was measured in each experiment by counting an aliquot of a sample (2 µl) of the radioligand preparation in a scintillation counter. Binding of [^{18}F]-**9** to human brain homogenates was assessed by incubating samples of insoluble fraction (1.25 µg insoluble protein/well) from AD subjects, with increasing concentrations of [^{18}F]-**9** (0.1nM-18 nM) for 1 h at 37 °C. Nonspecific binding was determined in parallel experiments in the presence of **5** (1 µM) as a competitor. Bound and free radioligand were separated by vacuum filtration through glass fiber 96-well filter plates (Millipore Multiscreen HTS FB filter plate, Cat# MSFBN6B50), followed by washes using ice cold assay buffer (3×150 µl). Glass fiber filters containing the bound ligand were mixed with Optiphase Supermix scintillation cocktail (150µl; PerkinElmer) and counted immediately. All data were obtained in triplicate and analyzed by curve fitting to a one-site binding model using nonlinear regression employing Graphpad Prism software (version 4.0) to determine K_d and B_{max} values. B_{max} values were calculated in pmol/gram wet weight of brain tissue. [13]

Human Serum Stability Analysis.

Human serum (Sigma Aldrich) was thawed. Serum (100µL) was taken, diluted with saline to 10%, and incubated with HPLC purified fraction of ^{18}F-**9** (100µCi each) at 37 °C for 30 min, 1h and 3h, respectively. Samples were periodically withdrawn, and analyzed on radio-HPLC (**SI-Figure 6**).

Autoradiography of Human Brain Sections and Immunohistochemistry Correlation

Autoradiography and immunohistochemistry (IHC) were performed as previously described[14] with minor modifications. Briefly, a sample of frozen AD frontal cortex was sectioned using a TBS Minotome PLUS Cryostat set at a thickness of 12 microns. The tissue sections were thaw-mounted onto Superfrost Plus (Fisherbrand 12-550-15) microscope slides and allowed to air dry for 10–15 min prior to storage at -80 °C. Adjacent sections were incubated in either 2 nM ^{18}F- **9** or 2 nM ^{18}F- **9** plus 1 µM cold **5** (20 ml/slide) for 60 min at room temperature. Sections were then washed at RT in 30 mM Tris-HCl pH 7.4 for 1 min, 70% ethanol/30 mM Tris-HCl for 2 min, 30% ethanol/30 mM Tris-HCl for 1 min and 30 mM Tris-HCl for 1 min. The sections were then dried and exposed to a phosphor imaging screen (BAS-TR 2025) for 45 min. Autoradiography images were obtained on GE Typhoon System FLA-9500 (Tokyo, Japan) at 25 µm resolution. Data was exported as linear 16-bit grayscale TIFF images and further processed by Adobe Photoshop CS3. For IHC, the same sections were blocked for 60 min at room temperature with 3% milk-0.25% Tween20-PBS buffer. After a 1 min wash in 0.5% milk-PBS-0.25% Tween 20, the sections were stained with monoclonal anti-Aβ antibody HJ 3.4 (1.7 µg/ml in 0.5% milk-PBS-0.25% Tween 20) at 4 °C overnight. The slides were washed 3 x 5 min with PBS-0.25% Tween 20, then dried and cover-slipped with DAPI Fluoromount-G (Cat# 0100-20, SouthernBiotech, Birmingham, AL). IHC images were obtained on a NanoZoomer 2.0-HT System (Olympus) at 20x resolution with DAPI and TRITC fluorescence filters using NDP.scan 2.5 software (Olympus).

Ex Vivo Autoradiography of Mice Brain Sections and Immunohistochemistry Correlation

Ex-vivo autoradiography was performed by tail vein injection of [^{18}F]-**9** (170 µCi, 8% ethanol in saline) in a 24-month old APP/PS1 transgenic (Tg) mouse and an age matched WT BL6 control female mouse.
The transgenic and WT mice were sacrificed at 30 min post injection. Brain tissue was removed and immediately frozen on powdered dry ice. Coronal sections (20 µm) were cut on a cryotstat (Leica), mounted on Superfrost Plus (Fisherbrand 12-550-15) microscope slides and allowed to air dry. The slides

were then exposed to a phosphor imaging screen (BAS-SR 2025) for 2 h before scanning on a Typhoon FLA-9500 system. The same sections were then immunostained with anti-Aβ antibody HJ 3.4 as described above. Autoradiography images were analyzed using MultiGauge software (Fuji, Tokyo, Japan). To quantify radiotracer accumulation, three target regions of interest (ROIs) (two in cortex and one in amygdala) were selected in Tg and WT sections where Aβ plaque density was high in Tg. Hypothalamus was selected as a reference ROI with low plaque density (The Mouse Brain in Stereotaxic Coordinates, 2nd edition).
The count intensity (expressed as quantitative light unit (QL)/pixel^2) was measured for each ROI after background subtraction, and target/reference ratios for Tg and WT brain were used to compare radiotracer uptake. The final figure was assembled in Adobe Photoshop CS3.

Biodistribution Studies.

All animal procedures were approved by the Washington University Animal Studies Committee (Protocol # 20150147; Expiration 7/31/18). Thus, biodistribution studies were carried out in "accordance" with the approved guidelines. Pharmacokinetics of ^{18}F-**9** in brain and other critical tissues of normal male 12 week old BL6 (WT; 28-36 g) mice were determined as previously described [15]. Briefly, ^{18}F-**9** (740 kBq) was dissolved in 100 µl saline containing 10% ethanol. All animals were anesthetized by isoflurane inhalation and injected with radiotracer ^{18}F-**9** (740 kBq, 100 µL) via bolus injection through a tail vein. Animals were sacrificed by cervical dislocation under anesthesia at 5, 30, 60, and 120 min after injection (n = 3 each). Blood samples were obtained by cardiac puncture, organs then harvested rapidly, and all tissue samples analyzed for γ-activity. Data (**SI-Table 1**) are expressed as the percentage injected dose (%ID) per gram of tissue (tissue kBq (injected kBq)$^{-1}$ (g tissue)$^{-1}$ x 100).

Multiphoton Imaging

All experimental protocols were approved by the Animal Studies Committee at Washington University (protocol #20140182). Thus, multiphoton imaging studies were carried out in "accordance" with the

approved guidelines. 15-month-old $APP^{+/-}/PS1^{+/-}$ mice were imaged using intravital multiphoton microscopy to assess pharmacokinetic profiles of the **5**. The thinned-skull cranial windows were prepared on the day prior to imaging, as previously described[16-18]. Briefly, mice were anesthetized under volatile isoflurane (2% induction, 1.5% maintenance), the scalp and periosteum were removed to expose the skull. A speed drill and microsurgical blade (Surgistar) were used to thin the skull until the skull window (1.5 mM in diameter) was transparent and displayed flexibility (approximately 10-20 µm thick). Animals were mounted on a custom-built stereotaxic apparatus and a small ring of molten bone wax was applied to the skull surrounding the perimeter of the window to create a water immersion chamber. The cranial window was centered under the objective lens on a two-photon microscope [LSM 510 META NLO system (Carl Zeiss Inc.) with a Cameleon Ti:Sapphire laser (Coherent Inc.)]. Prior to imaging, mice were intravenously injected with dextran-Texas Red conjugate to mark blood vessels (33mg/kg dissolved in PBS).

For imaging **5** labeled plaques, two-photon fluorescence was generated with 800 nm excitation, and fluorescent emission was detected in the range of 435-485nm. To image dextran-texas red labeled vessel, 800 nm was used for excitation, and 565-615nm for emission. A 10× water-immersion objective [numerical aperture (NA) = 0.33, Zeiss) was used. Time-lapse multiphoton imaging was initiated 5min before dye **5** (2 mg/kg dissolved in 20% DMSO in 80% propylene glycol) was injected. Dye **5** was intravenously injected over a 3-min infusion period (19). Three-dimensional volumes were acquired (by collecting a stack of x-y sections starting at the surface of the thinned skull to 100 microns deep into the cortex) for assessing pharmacokinetics of **5** at high resolution in brains of living mice.

MicroPET/CT Imaging.

All microPET/CT imaging studies were approved by the Washington University Animal Studies Committee (Protocol # 20150147; Expiration 7/31/18) and conducted in "accordance" with the approved guidelines. For imaging, female double transgenic mouse (BL6/APP/PS1, 18 months old, n =3) and a female wild-type mouse (BL6; 18 months old, n=3) were anesthetized with isoflurane (1.5 -2.5 %) in oxygen at flow rate of 1-

2 L/min via an induction chamber and maintained with a nose cone. Following anesthesia, the mice were secured with their heads in the center of the field of view, fixed in the scanner in prone head first position (HFP), and placed in an acrylic imaging tray. Micro-PET imaging was performed using Inveon PET/CT scanner (Siemens Medical Solutions) following intravenous tail-vein injection of HPLC-purified ^{18}F-**9** (5.032 MBq; 28 µl; 35% ethanol in saline) employing the catheter system in a slow bolus, followed by flushing with isotonic saline solution. PET dynamic data acquisition was performed over 1h starting immediately following injection of the tracer. The emission data were normalized, corrected for attenuation, scatter, and decay. Attenuation correction was obtained using the co-registered CT data. The image volume consisted of 256×256×159 voxels, with a size of 0.39×0.39×0.8 mm^3 per voxel for the Inveon scanner. For anatomical visualization, PET images were co-registered with CT images from an Inveon PET/CT scanner. For analysis, brain uptake (Bq/mL) was normalized to injected dose and weight of animals. ROIs were drawn within the brain excluding cerebellum, counts were normalized to %ID/g, and weight of the mice to generate SUV values. PET data were also analyzed using ReferenceLogan plots to obtain distribution volume ratios (DVR) for APP/PS1 and WT mice brains, using cerebellum as a reference region. All image data were processed and analyzed using Inveon Research Workspace 4.1 software (Siemens).

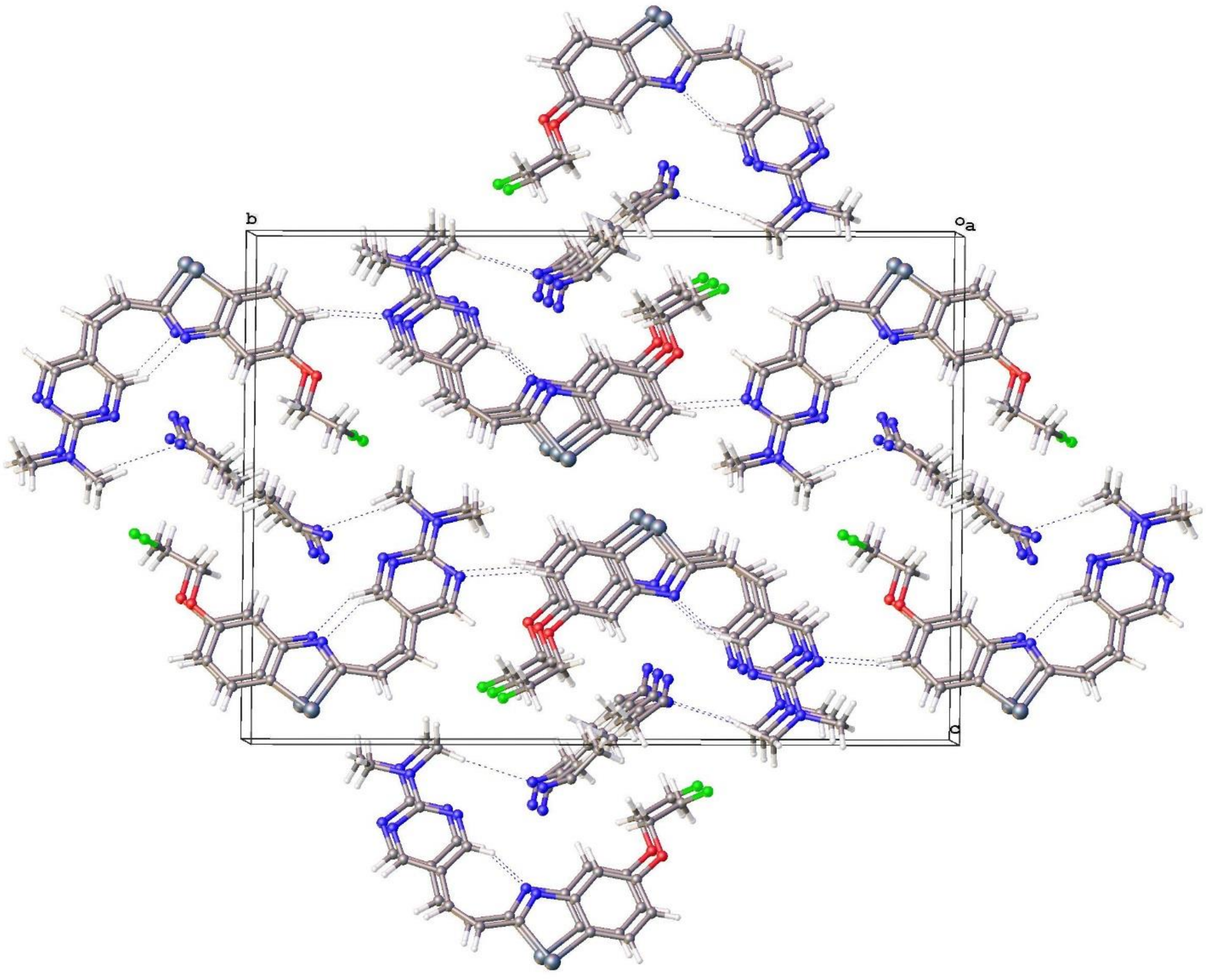

SI Figure 1. View of crystal packing of **5** with inter- and intramolecular H-bonding network.

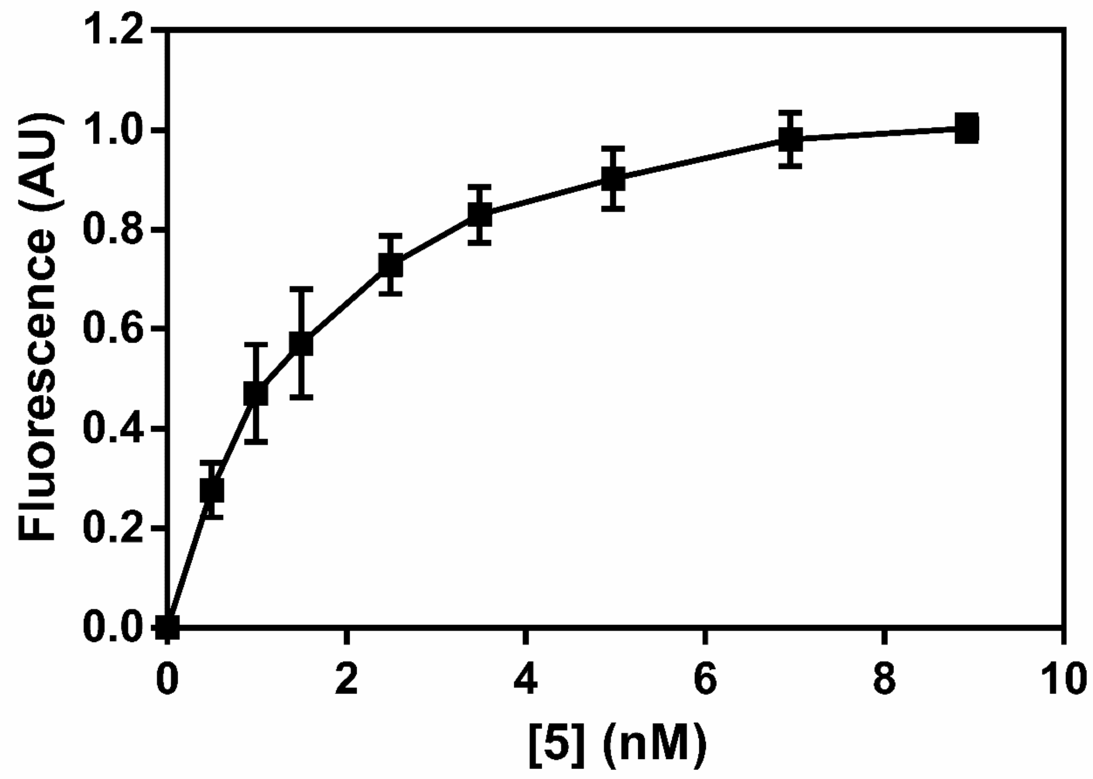

SI Figure 2. 5 (Fluselenamyl) was incubated in increasing concentrations with a fixed final concentration of $A\beta_{1\text{-}42}$ aggregates (100nM) at 37°C for 30 min in PBS. All measurements were made in triplicates; fluorescence of either **5** alone or fibrils at a given concentration was subtracted. The data were fitted into a single site model using GraphPad Prism, version 4.03 (GraphPad Software, San Diego,Ca, USA) and binding constant was found to be 1.58±0.05nM.

5 (Fluselenamyl) **Anti-Aβ Antibody mHJ3.4-AF568** **Merged**

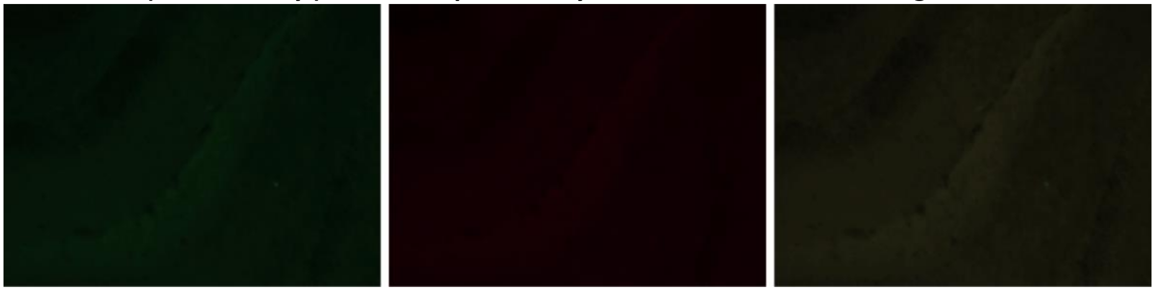

SI Figure 3. Staining of brain hippocampus sections of Bl6 (control) 10 months old mice, using either **5** or immunostained with HJ3.4 mouse monoclonal antibody and visualized by donkey-antimouse Alexa 568 (positive control). The slides were analyzed on a Zeiss LSM 5 PASCAL confocal system coupled to a Zeiss Axiovert 200 microscope. **Of Note:** Neither **5** nor Anti-Aβ antibody shows any staining of control mice tissue sections.

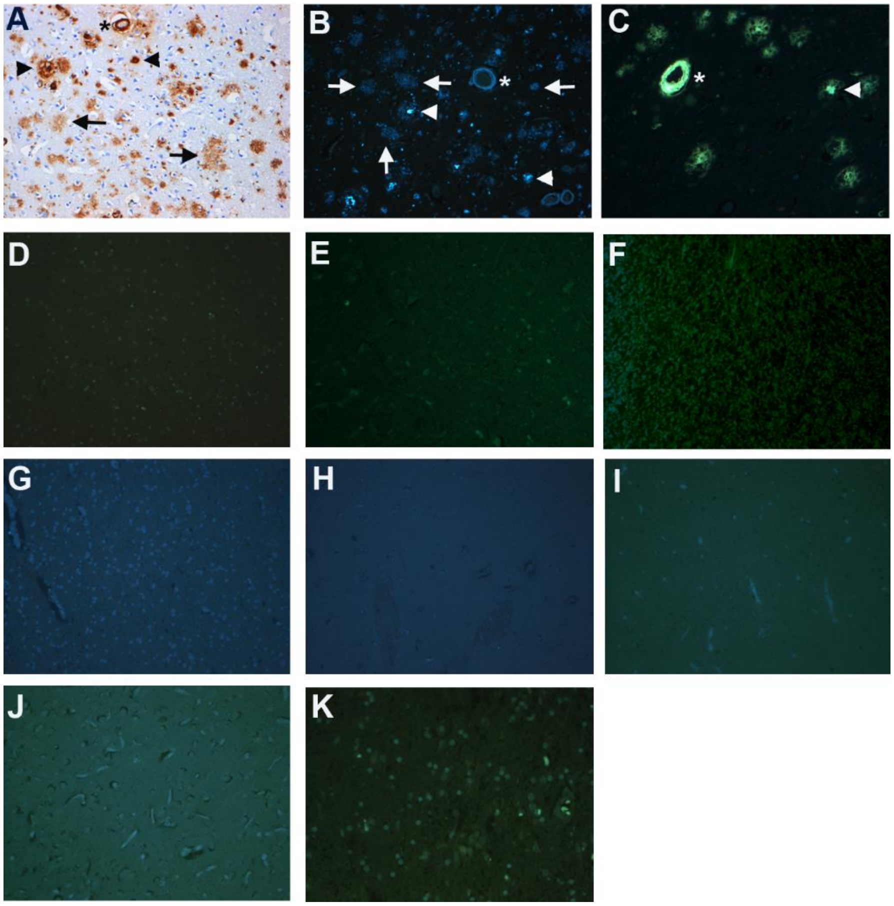

SI Figure 4A. In the Alzheimer's disease (AD) brain β-amyloid deposits are present as diffuse plaques (**Arrow**), more compact plaques (**Arrowhead**), and in the walls of some blood vessels (cerebral amyloid angiopathy, **Asterisk**) (beta-amyloid (10D5) immunohistochemistry). **B**: Fluselenamyl (**5)** detects compact plaques (**Arrowhead**), a proportion of diffuse plaques (**Arrow**), and vascular deposits (**Asterisk)**. **C**: Thioflavin S detects compact fibrillar beta-amyloid plaques and vascular amyloid. AI-187 does not detect any changes in the normal aging brain (**D**), or in the inclusions of other neurodegenerative diseases: **E**, Parkinson's disease (midbrain); **F**, multiple system atrophy (cerebellum); **G**, Huntington's disease (caudate nucleus); **H**, progressive supranuclear palsy (caudate nucleus); **I**, corticobasal degeneration; **J**, frontotemporal lobar degeneration with TDP-43 proteinopathy; and **K**, amyotrophic lateral sclerosis with TDP-43 proteinopathy. **A** to **D**, **F** to **J**, **Magnification**: 10X; **E** and **K**, **Magnification:** 20X.

Disease	Brain Area	Block#	Fluselenamyl (5)	Positive Control (antibody)
Alzheimer's disease + CAA	Frontal Lobe	L1	Positive (diffuse and compact Aβ Plaques) + CAA	10D5
Normal control	Frontal Lobe	L1	Negative	10D5
Parkinson's disease	Medulla	L12	Negative	pSYN
Multiple system atrophy	Cerebellum	L14	Negative	pSYN
Huntington's disease	Striatum	L6	Negative	1C2
Progressive supranuclear palsy	Striatum	L6	Negative	PHF1
Corticobasal degeneration	Frontal Lobe	L1	Negative	PHF1
FLTD with TDP proteinopathy	Frontal Lobe	L1	Negative	pTDP
ALS with TDP proteinopathy	Spinal Cord	L14	Negative	pTDP

SI Figure 4B. Specificity of Fluselenamyl (5) for Aβ plaques in Alzheimer's disease compared with biomarkers of other neurodegenerative diseases: Columns: **1-5** (left-Right). Human brain areas (used for obtaining tissue sections; **2rd Column**); results for **Fluselenamyl (5)** (**4th Column**), and antibodies (positive controls) used to confirm diseases in tissue sections (**5th Column**). CAA; Cerebral amyloid angiopathy.

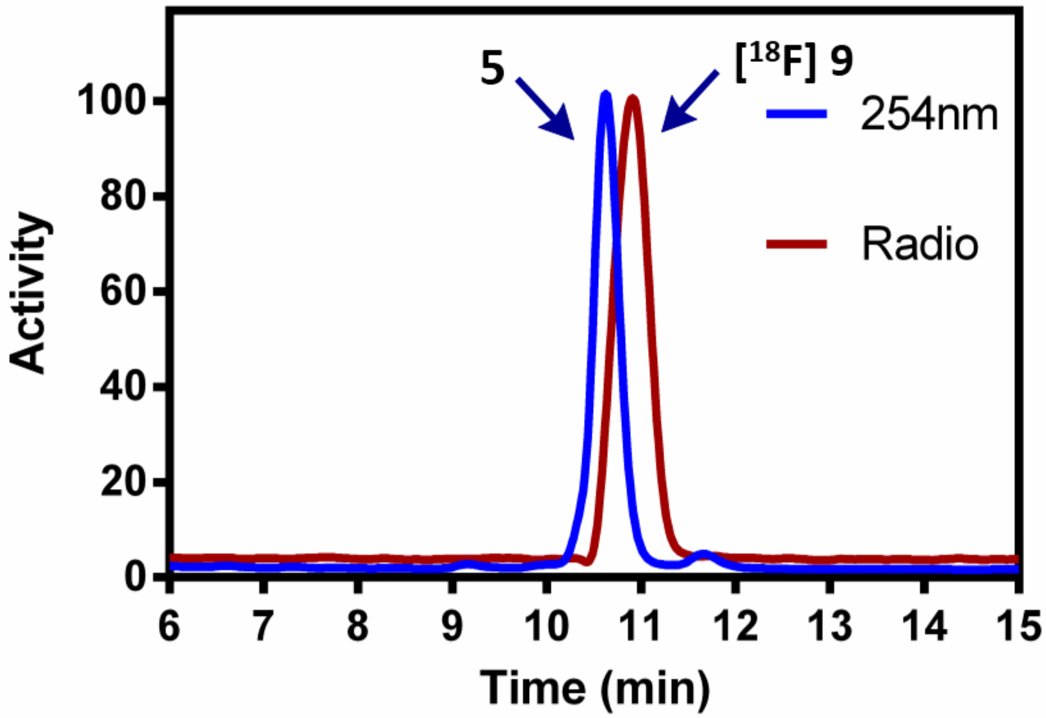

SI Figure 5. Radio-HPLC data of purified [^{18}F]-**9**, an Aβ-targeted probe, spiked with an unlabeled counterpart (**5**) for characterization of the radiotracer.

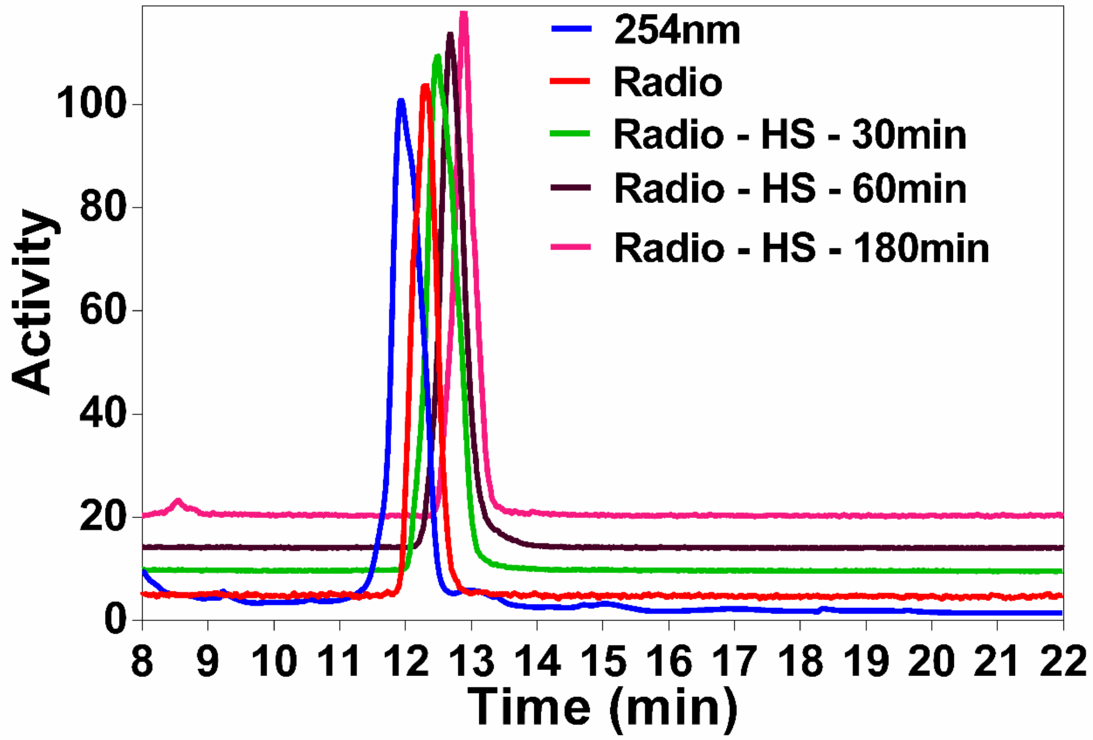

SI Figure 6. Stability in Human Serum. Radio-HPLC of ^{18}F-**9** (red) spiked with cold (blue), and following incubation (green, pink, and purple) with human serum. Peaks are offset for clarity. Note stability of the radiotracer for 3h in serum.

SI, Table 1. Biodistribution Data (%ID/g) of [^{18}F]-**9** in FVB mice (n = 3).

Organ (% ID/g)	2	60	120 min
Blood	4.79 ± 0.33	2.27 ± 0.06	2.16 ± 0.03
Brain	8.86± 0.32	1.80 ± 0.02	1.66 ± 0.01
Bone	1.15 ± 0.15	3.88 ± 1.48	6.08 ± 0.85
Liver	35.44 ± 2.52	8.64 ± 0.15	8.13 ± 0.12
Kidney	14.93± 0.85	2.52 ± 0.06	2.12 ± 0.09
Heart	8.56± 0.78	2.15 ± 0.02	1.92 ± 0.05
Lung	10.07± 0.74	2.23 ± 0.11	1.98 ± 0.05

Table 2. Crystal data and structure refinement parameters for **5**.

Identification code	v18213t5/rt/X8	
Empirical formula	$C_{19} H_{20} F N5 O Se$	
Formula weight	432.36	
Temperature	296(2) K	
Wavelength	0.71073 Å	
Crystal system	Monoclinic	
Space group	$P 2_1/n$	
Unit cell dimensions	a = 4.1693(5) Å	α= 90°.
	b = 25.146(3) Å	β= 93.403(7)°.
	c = 18.0584(18) Å	γ = 90°.
Volume	1889.9(4) $Å^3$	
Z	4	
Density (calculated)	1.520 Mg/m^3	
Absorption coefficient	2.016 mm^{-1}	
F(000)	880	
Crystal size	0.594 x 0.076 x 0.054 mm^3	
Theta range for data collection	1.975 to 24.997°.	
Index ranges	-4≤h≤4, 0≤k≤29, 0≤l≤21	
Reflections collected	3339	
Independent reflections	3339 [R(int) = 0.0405]	
Completeness to theta = 25.000°	98.1 %	
Absorption correction	Semi-empirical from equivalents	
Max. and min. transmission	0.745160 and 0.590024	
Refinement method	Full-matrix least-squares on F^2	
Data / restraints / parameters	3339 / 388 / 249	
Goodness-of-fit on F^2	1.061	
Final R indices [I>2sigma(I)]	R1 = 0.0634, wR2 = 0.1465	
R indices (all data)	R1 = 0.1156, wR2 = 0.1710	
Extinction coefficient	n/a	
Largest diff. peak and hole	0.582 and -0.396 e.$Å^{-3}$	

Table 3. Atomic coordinates (x 10^4) and equivalent isotropic displacement parameters (Å^{2}x 10^3) for **5**. U(eq) is defined as one third of the trace of the orthogonalized U^{ij} tensor.

	x	y	z	U(eq)
Se(1)	2849(2)	746(1)	750(1)	55(1)
F(1)	10316(14)	-1529(2)	4026(3)	86(2)
O(1)	9116(14)	-878(2)	2768(3)	58(2)
N(1)	6337(16)	934(2)	2050(3)	47(2)
N(2)	9956(16)	2920(2)	3300(3)	52(2)
N(3)	10726(16)	1992(2)	3552(3)	53(2)
N(4)	13016(16)	2640(2)	4328(3)	57(2)
C(1)	4632(19)	187(3)	1318(4)	44(2)
C(2)	4470(20)	-358(3)	1193(4)	52(2)
C(3)	6009(18)	-693(3)	1683(4)	49(2)
C(4)	7709(19)	-498(3)	2319(4)	46(2)
C(5)	7881(19)	40(2)	2462(4)	45(2)
C(6)	6261(18)	386(2)	1948(3)	40(2)
C(7)	4753(18)	1189(3)	1520(4)	46(2)
C(8)	4320(20)	1754(3)	1424(4)	59(2)
C(9)	5427(19)	2179(3)	1817(4)	53(2)
C(10)	7463(18)	2265(2)	2477(4)	45(2)
C(11)	8140(20)	2788(3)	2704(4)	52(2)
C(12)	11257(18)	2515(3)	3706(4)	44(2)
C(13)	8942(19)	1882(3)	2949(4)	50(2)
C(14)	13780(20)	3190(3)	4497(4)	70(3)
C(15)	14350(20)	2234(3)	4820(4)	72(3)
C(16)	10974(18)	-716(3)	3415(4)	55(2)
C(17)	12570(20)	-1189(3)	3773(5)	70(2)
N(1S')	3130(110)	900(10)	3731(19)	226(15)
C(1S')	5110(110)	811(10)	4140(30)	226(15)
C(2S')	6370(110)	382(10)	4571(15)	226(15)
N(1S)	1760(130)	924(15)	4191(19)	250(20)
C(1S)	2440(160)	518(15)	4358(19)	250(20)
C(2S)	2700(130)	128(13)	4910(20)	250(20)

Table 4. Bond lengths [Å] and angles [°] for **5**.

Se(1)-C(1)	1.868(7)
Se(1)-C(7)	1.917(7)
F(1)-C(17)	1.370(9)
O(1)-C(4)	1.364(8)
O(1)-C(16)	1.423(9)
N(1)-C(7)	1.299(8)
N(1)-C(6)	1.389(8)
N(2)-C(11)	1.322(9)
N(2)-C(12)	1.349(8)
N(3)-C(13)	1.310(8)
N(3)-C(12)	1.361(8)
N(4)-C(12)	1.342(9)
N(4)-C(15)	1.444(9)
N(4)-C(14)	1.447(9)
C(1)-C(6)	1.383(9)
C(1)-C(2)	1.389(9)
C(2)-C(3)	1.358(10)
C(2)-H(2)	0.9300
C(3)-C(4)	1.401(10)
C(3)-H(3)	0.9300
C(4)-C(5)	1.379(9)
C(5)-C(6)	1.415(9)
C(5)-H(5)	0.9300
C(7)-C(8)	1.441(9)
C(8)-C(9)	1.348(9)
C(8)-H(8)	0.9300
C(9)-C(10)	1.437(10)
C(9)-H(9)	0.9300
C(10)-C(11)	1.400(9)
C(10)-C(13)	1.406(9)
C(11)-H(11)	0.9300
C(13)-H(13)	0.9300
C(14)-H(14A)	0.9600
C(14)-H(14B)	0.9600

C(14)-H(14C)	0.9600
C(15)-H(15A)	0.9600
C(15)-H(15B)	0.9600
C(15)-H(15C)	0.9600
C(16)-C(17)	1.491(10)
C(16)-H(16A)	0.9700
C(16)-H(16B)	0.9700
C(17)-H(17A)	0.9700
C(17)-H(17B)	0.9700
N(1S')-C(1S')	1.103(10)
C(1S')-C(2S')	1.410(10)
C(2S')-H(2S1)	0.9600
C(2S')-H(2S2)	0.9600
C(2S')-H(2S3)	0.9600
N(1S)-C(1S)	1.098(10)
C(1S)-C(2S)	1.398(10)
C(2S)-H(2S4)	0.9600
C(2S)-H(2S5)	0.9600
C(2S)-H(2S6)	0.9600

C(1)-Se(1)-C(7)	84.6(3)
C(4)-O(1)-C(16)	118.8(5)
C(7)-N(1)-C(6)	112.7(6)
C(11)-N(2)-C(12)	116.5(6)
C(13)-N(3)-C(12)	116.8(6)
C(12)-N(4)-C(15)	121.4(6)
C(12)-N(4)-C(14)	120.3(6)
C(15)-N(4)-C(14)	118.3(6)
C(6)-C(1)-C(2)	120.6(7)
C(6)-C(1)-Se(1)	109.8(5)
C(2)-C(1)-Se(1)	129.6(6)
C(3)-C(2)-C(1)	119.3(7)
C(3)-C(2)-H(2)	120.3
C(1)-C(2)-H(2)	120.3
C(2)-C(3)-C(4)	120.9(6)
C(2)-C(3)-H(3)	119.6

C(4)-C(3)-H(3)	119.6
O(1)-C(4)-C(5)	124.1(6)
O(1)-C(4)-C(3)	114.8(6)
C(5)-C(4)-C(3)	121.0(7)
C(4)-C(5)-C(6)	117.6(6)
C(4)-C(5)-H(5)	121.2
C(6)-C(5)-H(5)	121.2
C(1)-C(6)-N(1)	118.3(6)
C(1)-C(6)-C(5)	120.5(6)
N(1)-C(6)-C(5)	121.1(6)
N(1)-C(7)-C(8)	129.1(7)
N(1)-C(7)-Se(1)	114.6(5)
C(8)-C(7)-Se(1)	116.3(5)
C(9)-C(8)-C(7)	132.9(7)
C(9)-C(8)-H(8)	113.5
C(7)-C(8)-H(8)	113.5
C(8)-C(9)-C(10)	136.3(7)
C(8)-C(9)-H(9)	111.9
C(10)-C(9)-H(9)	111.9
C(11)-C(10)-C(13)	113.1(6)
C(11)-C(10)-C(9)	118.9(6)
C(13)-C(10)-C(9)	128.0(6)
N(2)-C(11)-C(10)	124.7(7)
N(2)-C(11)-H(11)	117.6
C(10)-C(11)-H(11)	117.6
N(4)-C(12)-N(2)	117.3(6)
N(4)-C(12)-N(3)	118.2(6)
N(2)-C(12)-N(3)	124.3(6)
N(3)-C(13)-C(10)	124.5(6)
N(3)-C(13)-H(13)	117.8
C(10)-C(13)-H(13)	117.8
N(4)-C(14)-H(14A)	109.5
N(4)-C(14)-H(14B)	109.5
H(14A)-C(14)-H(14B)	109.5
N(4)-C(14)-H(14C)	109.5
H(14A)-C(14)-H(14C)	109.5

H(14B)-C(14)-H(14C)	109.5
N(4)-C(15)-H(15A)	109.5
N(4)-C(15)-H(15B)	109.5
H(15A)-C(15)-H(15B)	109.5
N(4)-C(15)-H(15C)	109.5
H(15A)-C(15)-H(15C)	109.5
H(15B)-C(15)-H(15C)	109.5
O(1)-C(16)-C(17)	109.6(6)
O(1)-C(16)-H(16A)	109.8
C(17)-C(16)-H(16A)	109.8
O(1)-C(16)-H(16B)	109.8
C(17)-C(16)-H(16B)	109.8
H(16A)-C(16)-H(16B)	108.2
F(1)-C(17)-C(16)	110.2(7)
F(1)-C(17)-H(17A)	109.6
C(16)-C(17)-H(17A)	109.6
F(1)-C(17)-H(17B)	109.6
C(16)-C(17)-H(17B)	109.6
H(17A)-C(17)-H(17B)	108.1
N(1S')-C(1S')-C(2S')	140(3)
C(1S')-C(2S')-H(2S1)	109.5
C(1S')-C(2S')-H(2S2)	109.5
H(2S1)-C(2S')-H(2S2)	109.5
C(1S')-C(2S')-H(2S3)	109.5
H(2S1)-C(2S')-H(2S3)	109.5
H(2S2)-C(2S')-H(2S3)	109.5
C(2S)-C(1S)-N(1S)	149(3)
C(1S)-C(2S)-H(2S4)	109.5
C(1S)-C(2S)-H(2S5)	109.5
H(2S4)-C(2S)-H(2S5)	109.5
C(1S)-C(2S)-H(2S6)	109.5
H(2S4)-C(2S)-H(2S6)	109.5
H(2S5)-C(2S)-H(2S6)	109.5

Symmetry transformations used to generate equivalent atoms:

Table 5. Anisotropic displacement parameters ($Å^2 x 10^3$) for **5**. The anisotropic displacement factor exponent takes the form: $-2▯^2[h^2 a^{*2}U^{11} + ... + 2 h k a^* b^* U^{12}]$

	U^{11}	U^{22}	U^{33}	U^{23}	U^{13}	U^{12}
Se(1)	63(1)	56(1)	43(1)	-4(1)	-8(1)	-3(1)
F(1)	86(4)	70(3)	101(4)	36(3)	-13(3)	-5(3)
O(1)	71(4)	40(3)	61(3)	4(2)	-4(3)	0(2)
N(1)	56(4)	38(3)	46(3)	-6(2)	-6(3)	1(3)
N(2)	65(4)	38(3)	53(3)	0(2)	-11(3)	3(3)
N(3)	69(4)	40(3)	50(3)	3(2)	-13(3)	-3(3)
N(4)	69(5)	46(3)	55(3)	-3(2)	-17(3)	-6(3)
C(1)	48(5)	47(3)	38(3)	-4(2)	6(3)	-4(3)
C(2)	60(5)	52(3)	44(4)	-9(3)	6(4)	-10(3)
C(3)	59(5)	40(3)	48(3)	-9(3)	7(3)	-10(3)
C(4)	49(5)	39(3)	52(3)	-1(3)	5(3)	-8(3)
C(5)	54(5)	38(3)	42(3)	-6(2)	-2(3)	-5(3)
C(6)	47(4)	40(3)	32(3)	-6(2)	4(3)	-7(3)
C(7)	46(5)	49(3)	43(4)	0(3)	-2(3)	2(3)
C(8)	79(6)	48(3)	48(4)	-2(3)	-13(4)	7(3)
C(9)	69(5)	39(3)	51(4)	1(3)	-5(4)	5(3)
C(10)	55(5)	34(3)	45(3)	4(2)	-2(3)	4(3)
C(11)	60(5)	41(3)	55(4)	3(3)	-1(4)	3(3)
C(12)	48(5)	40(3)	45(3)	0(2)	5(3)	-1(3)
C(13)	68(5)	38(3)	44(3)	-3(3)	-7(3)	5(3)
C(14)	85(7)	52(4)	73(5)	-11(4)	-8(5)	-4(4)
C(15)	83(7)	64(5)	66(5)	11(4)	-18(5)	-3(5)
C(16)	50(5)	53(4)	61(4)	2(3)	4(3)	-3(4)
C(17)	66(6)	59(4)	83(5)	12(4)	-6(4)	-2(4)
N(1S')	380(40)	80(10)	210(30)	16(12)	-30(20)	43(17)
C(1S')	380(40)	80(10)	210(30)	16(12)	-30(20)	43(17)
C(2S')	380(40)	80(10)	210(30)	16(12)	-30(20)	43(17)
N(1S)	370(40)	200(30)	170(20)	-130(20)	-100(30)	70(30)
C(1S)	370(40)	200(30)	170(20)	-130(20)	-100(30)	70(30)
C(2S)	370(40)	200(30)	170(20)	-130(20)	-100(30)	70(30)

Table 6. Hydrogen coordinates (x 10^4) and isotropic displacement parameters ($Å^2$x 10^3)
For **5**.

	x	y	z	U(eq)
H(2)	3318	-491	776	62
H(3)	5934	-1058	1597	59
H(5)	9022	171	2881	54
H(8)	3026	1844	1006	71
H(9)	4680	2497	1609	64
H(11)	7238	3062	2415	63
H(13)	8632	1525	2826	60
H(14A)	15302	3206	4917	106
H(14B)	11860	3376	4609	106
H(14C)	14693	3353	4077	106
H(15A)	13869	1890	4612	108
H(15B)	13430	2264	5293	108
H(15C)	16637	2279	4882	108
H(16A)	9596	-547	3759	66
H(16B)	12584	-460	3282	66
H(17A)	13835	-1371	3417	84
H(17B)	14010	-1074	4184	84
H(2S1)	4645	196	4789	339
H(2S2)	7842	513	4956	339
H(2S3)	7469	143	4258	339
H(2S4)	3324	-203	4698	381
H(2S5)	665	86	5126	381
H(2S6)	4291	234	5289	381

Table 7. Torsion angles [°] for **5**.

C(7)-Se(1)-C(1)-C(6)	-0.1(6)
C(7)-Se(1)-C(1)-C(2)	179.0(8)
C(6)-C(1)-C(2)-C(3)	-1.9(11)
Se(1)-C(1)-C(2)-C(3)	179.1(6)
C(1)-C(2)-C(3)-C(4)	1.0(11)
C(16)-O(1)-C(4)-C(5)	-2.1(11)
C(16)-O(1)-C(4)-C(3)	178.1(7)
C(2)-C(3)-C(4)-O(1)	179.4(7)
C(2)-C(3)-C(4)-C(5)	-0.3(11)
O(1)-C(4)-C(5)-C(6)	-179.2(7)
C(3)-C(4)-C(5)-C(6)	0.5(11)
C(2)-C(1)-C(6)-N(1)	-179.7(6)
Se(1)-C(1)-C(6)-N(1)	-0.5(9)
C(2)-C(1)-C(6)-C(5)	2.1(11)
Se(1)-C(1)-C(6)-C(5)	-178.7(6)
C(7)-N(1)-C(6)-C(1)	1.1(11)
C(7)-N(1)-C(6)-C(5)	179.3(7)
C(4)-C(5)-C(6)-C(1)	-1.4(11)
C(4)-C(5)-C(6)-N(1)	-179.5(7)
C(6)-N(1)-C(7)-C(8)	-178.9(7)
C(6)-N(1)-C(7)-Se(1)	-1.1(9)
N(1)-C(7)-C(8)-C(9)	1.9(16)
Se(1)-C(7)-C(8)-C(9)	-176.0(8)
C(7)-C(8)-C(9)-C(10)	0.8(18)
C(8)-C(9)-C(10)-C(11)	178.1(9)
C(8)-C(9)-C(10)-C(13)	-2.1(16)
C(12)-N(2)-C(11)-C(10)	0.7(12)
C(13)-C(10)-C(11)-N(2)	-0.2(12)
C(9)-C(10)-C(11)-N(2)	179.7(8)
C(15)-N(4)-C(12)-N(2)	176.4(7)
C(14)-N(4)-C(12)-N(2)	-7.4(11)
C(15)-N(4)-C(12)-N(3)	0.6(12)
C(14)-N(4)-C(12)-N(3)	176.9(6)
C(11)-N(2)-C(12)-N(4)	-177.6(7)

C(11)-N(2)-C(12)-N(3)	-2.2(11)
C(13)-N(3)-C(12)-N(4)	178.5(7)
C(13)-N(3)-C(12)-N(2)	3.1(11)
C(12)-N(3)-C(13)-C(10)	-2.6(12)
C(11)-C(10)-C(13)-N(3)	1.3(11)
C(9)-C(10)-C(13)-N(3)	-178.6(8)
C(4)-O(1)-C(16)-C(17)	-173.7(7)
O(1)-C(16)-C(17)-F(1)	-65.1(8)

Symmetry transformations used to generate equivalent atoms:

Table 8. Hydrogen bonds for **5** [Å and °].

D-H...A	d(D-H)	d(H...A)	d(D...A)	<(DHA)
C(3)-H(3)...N(2)#1	0.93	2.61	3.511(9)	164.5
C(13)-H(13)...N(1)	0.93	2.22	3.048(9)	147.6
C(15)-H(15A)...N(1S)#2	0.96	2.68	3.63(4)	170.5

Symmetry transformations used to generate equivalent atoms:

#1 -x+3/2,y-1/2,-z+1/2 #2 x+1,y,z

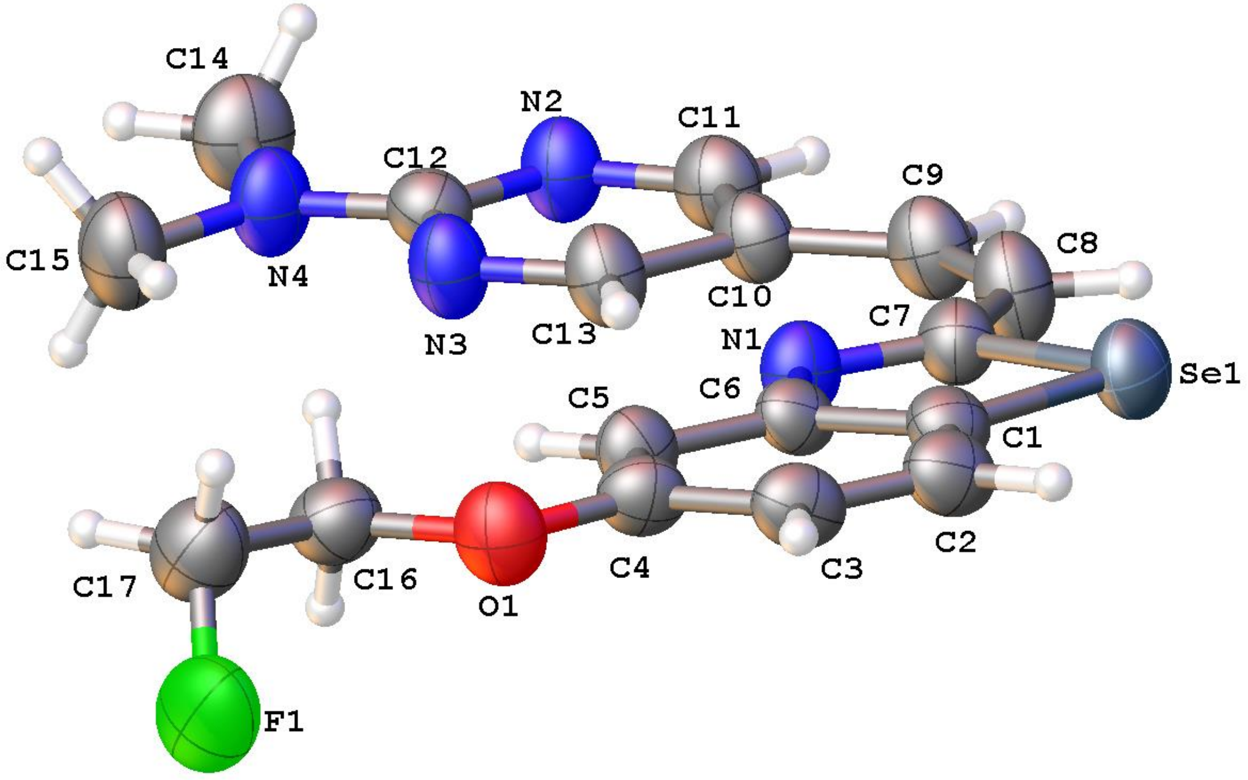

SI Figure 7. Projection view of **5** with 30% probability ellipsoids and disordered solvent acetonitrile omitted.

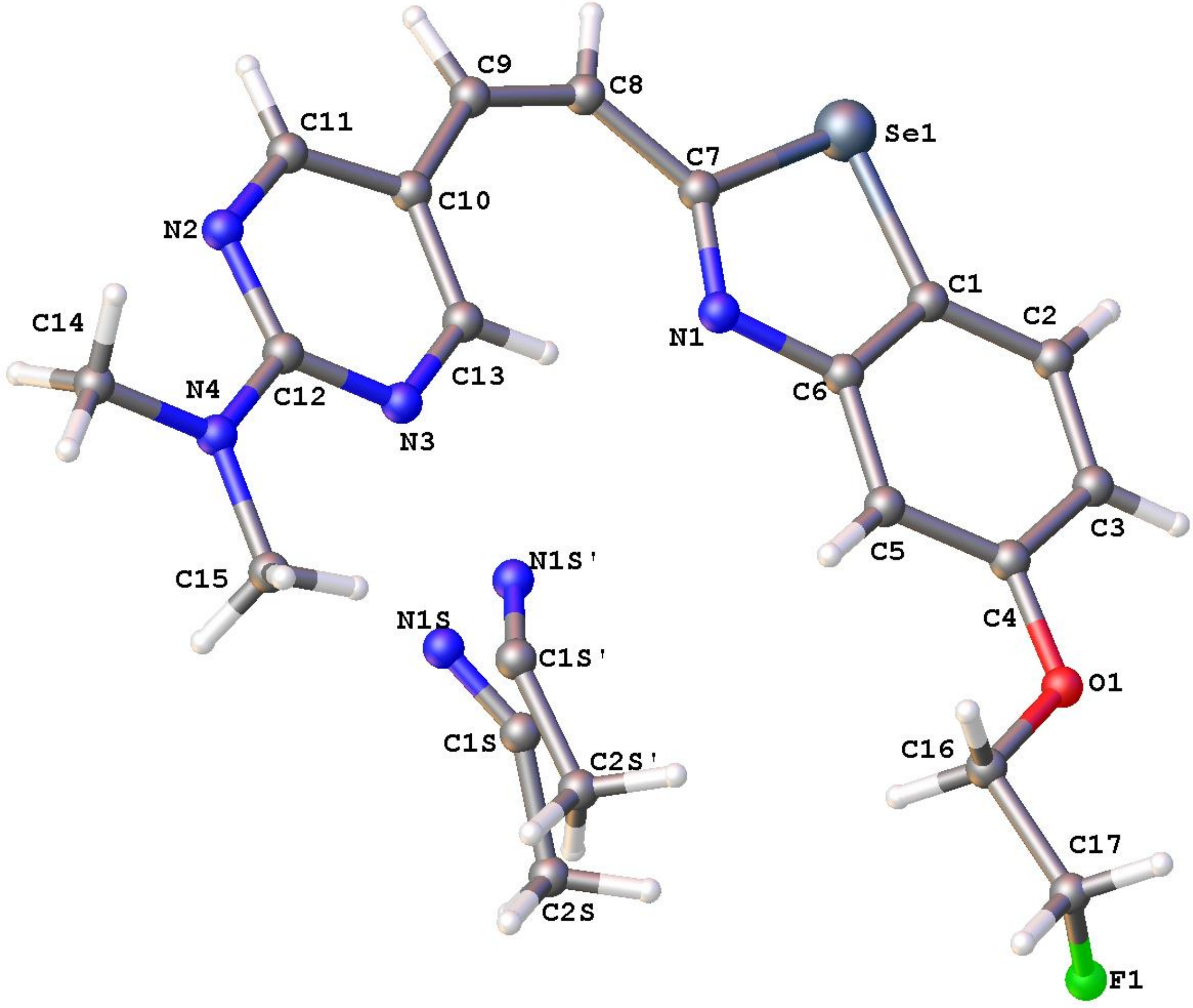

SI Figure 8. Projection view of **5** with 30% probability ellipsoids and disordered solvent acetonitrile included.

NMR Spectral Data

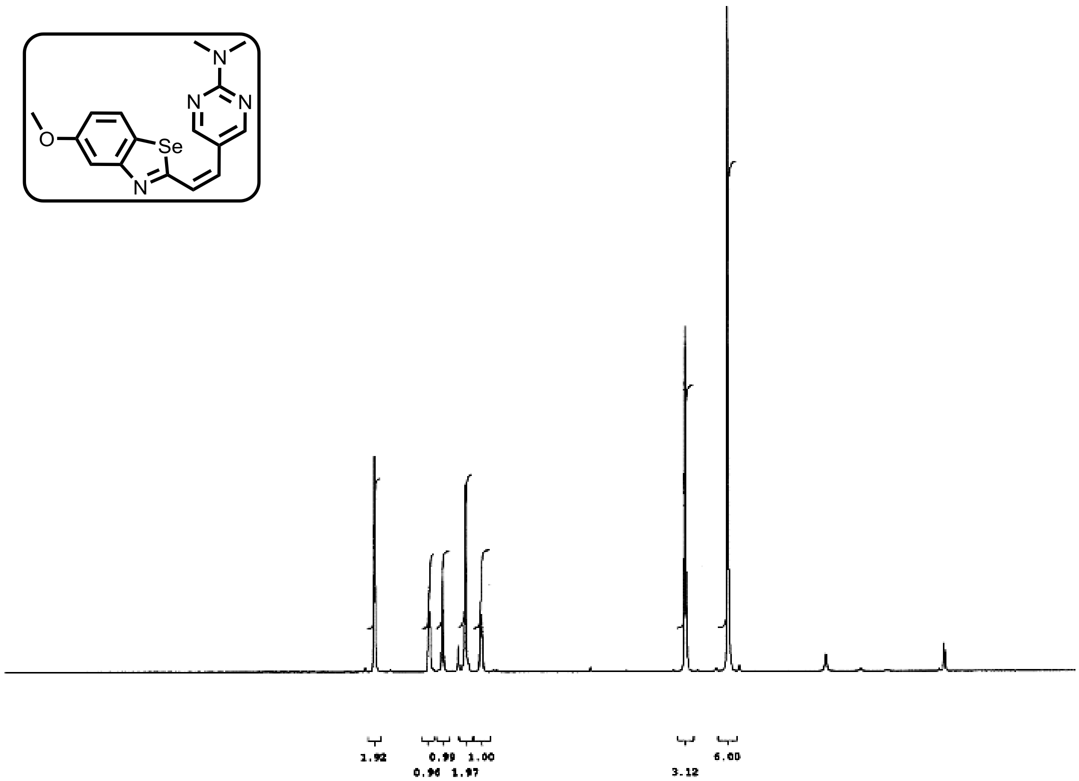

^{1}H spectrum of compound 3

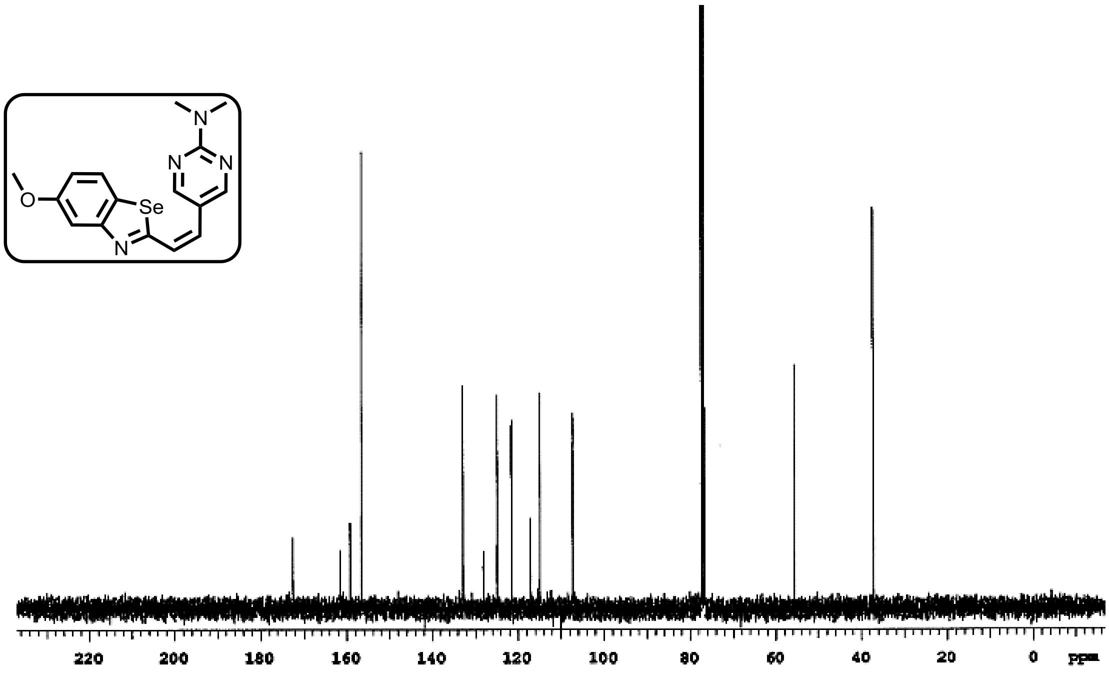

^{13}C NMR Spectrum of compound 3

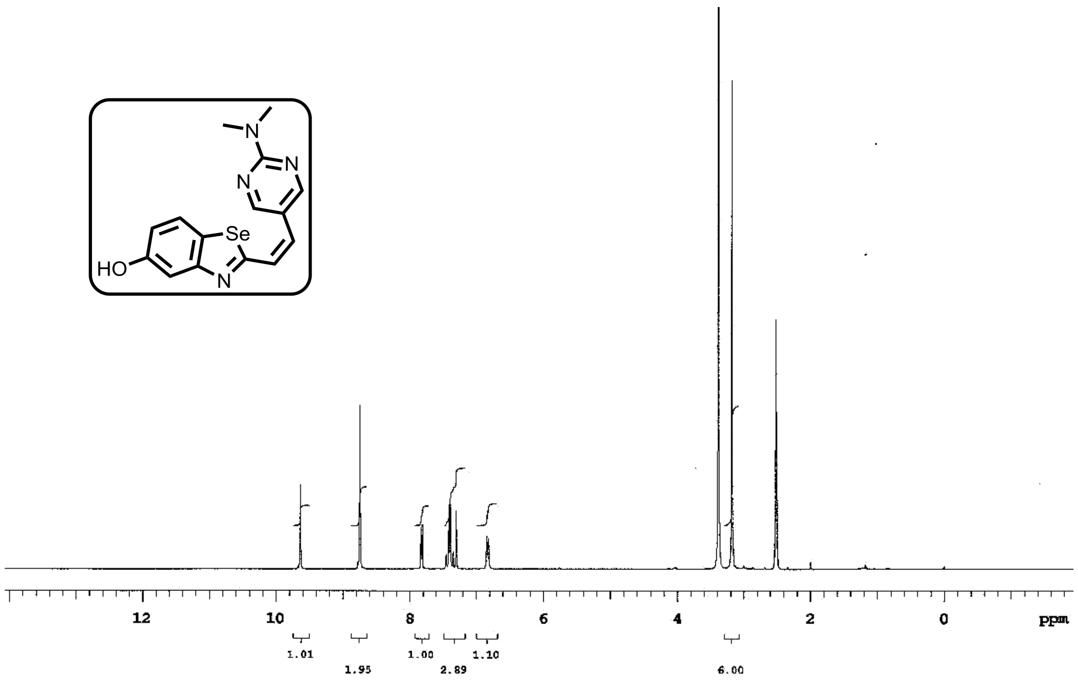

^{1}H spectrum of compound 4

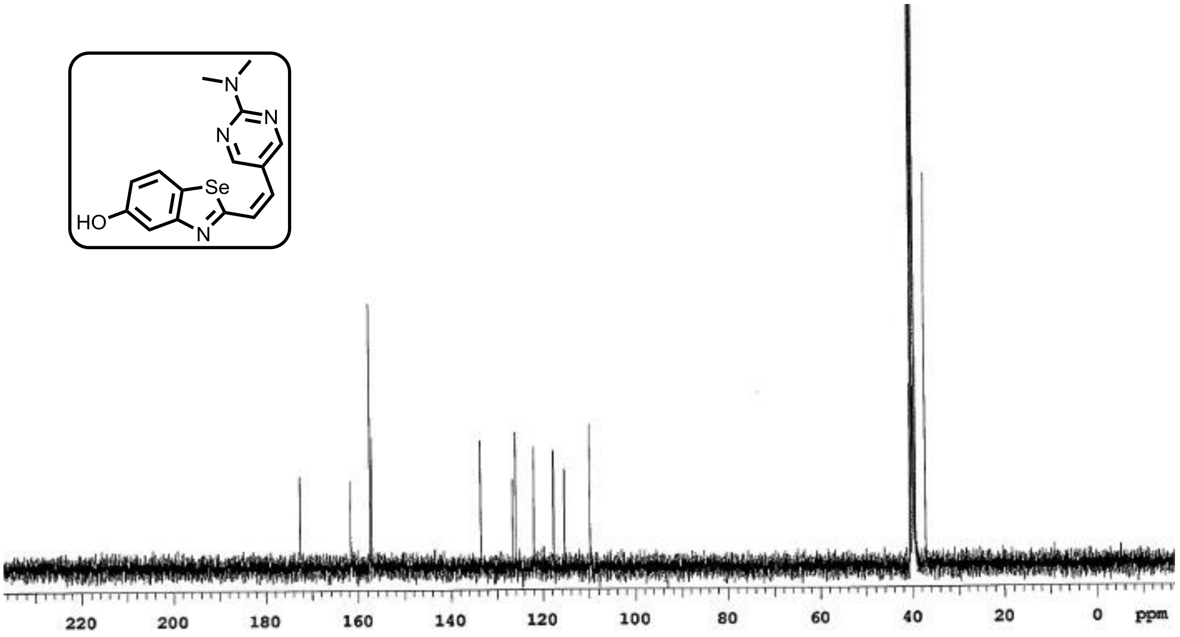

^{13}C NMR Spectrum of compound 4

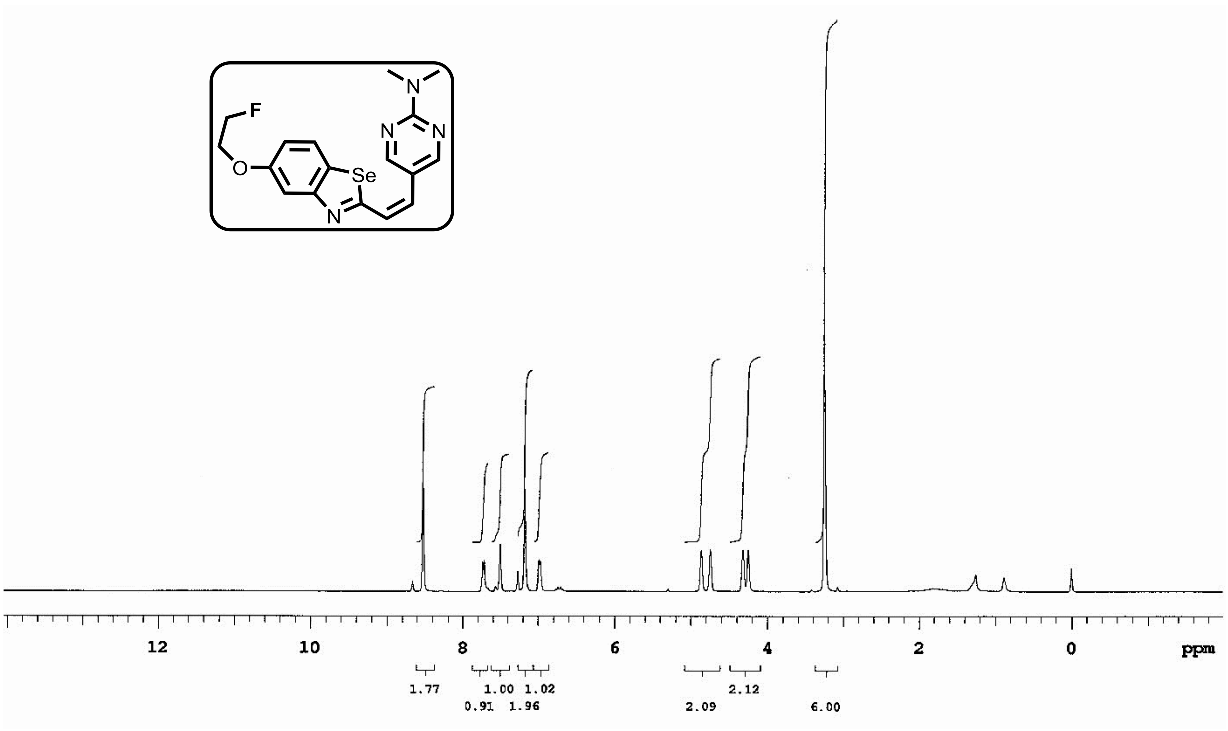

^{1}H spectrum of compound 5

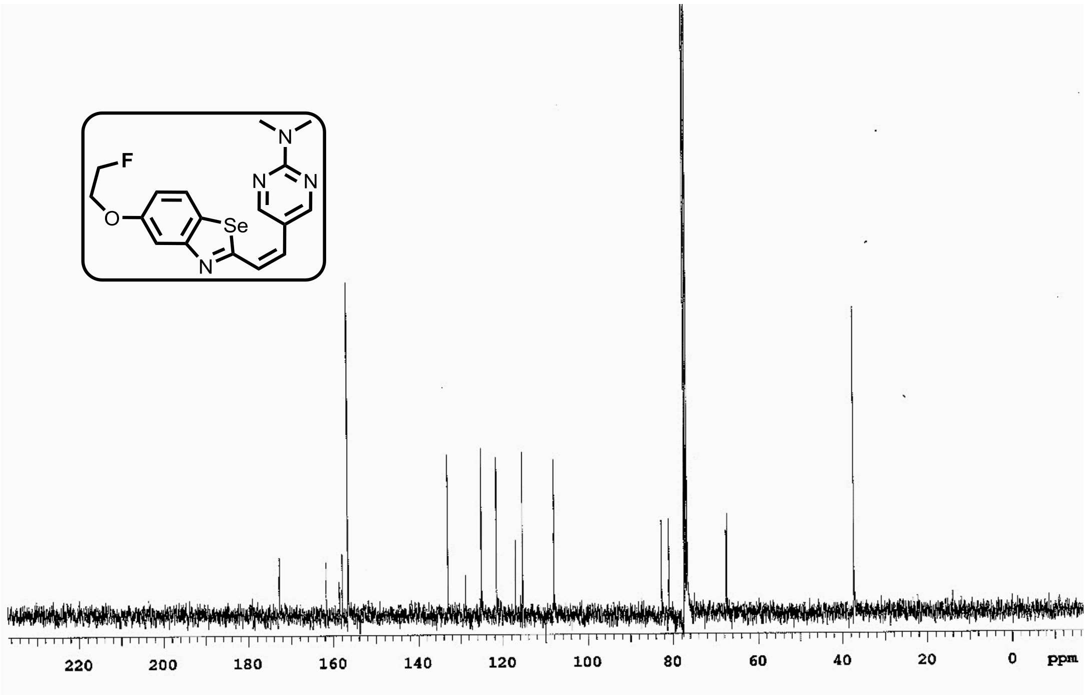

^{13}C NMR Spectrum of compound 5

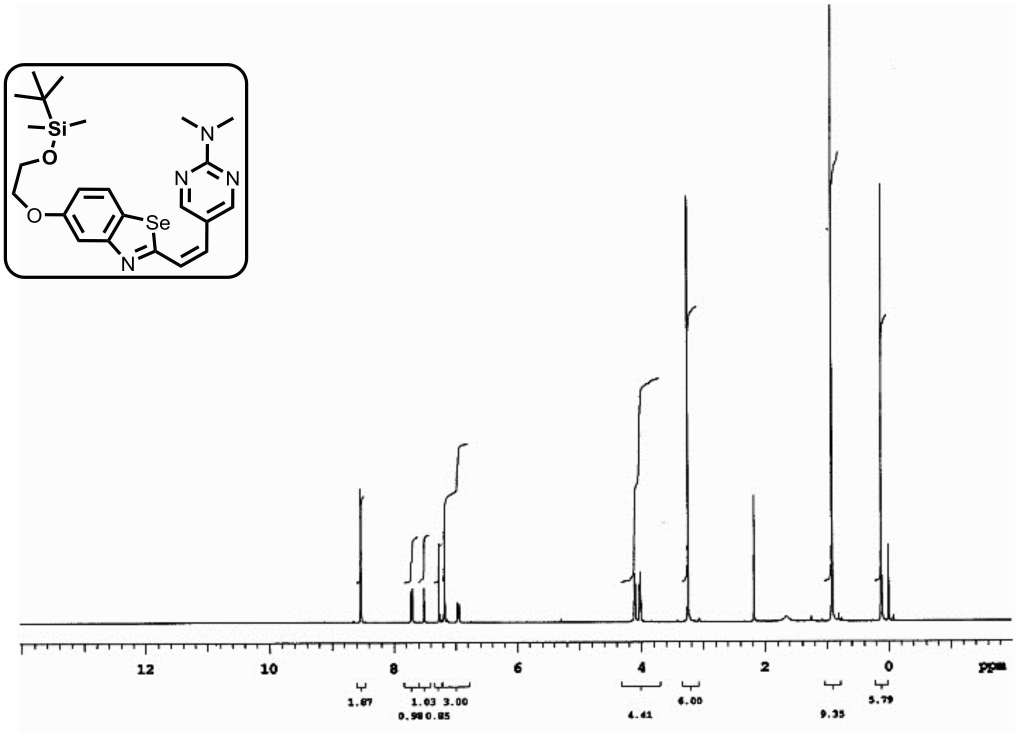

^{1}H spectrum of compound 6

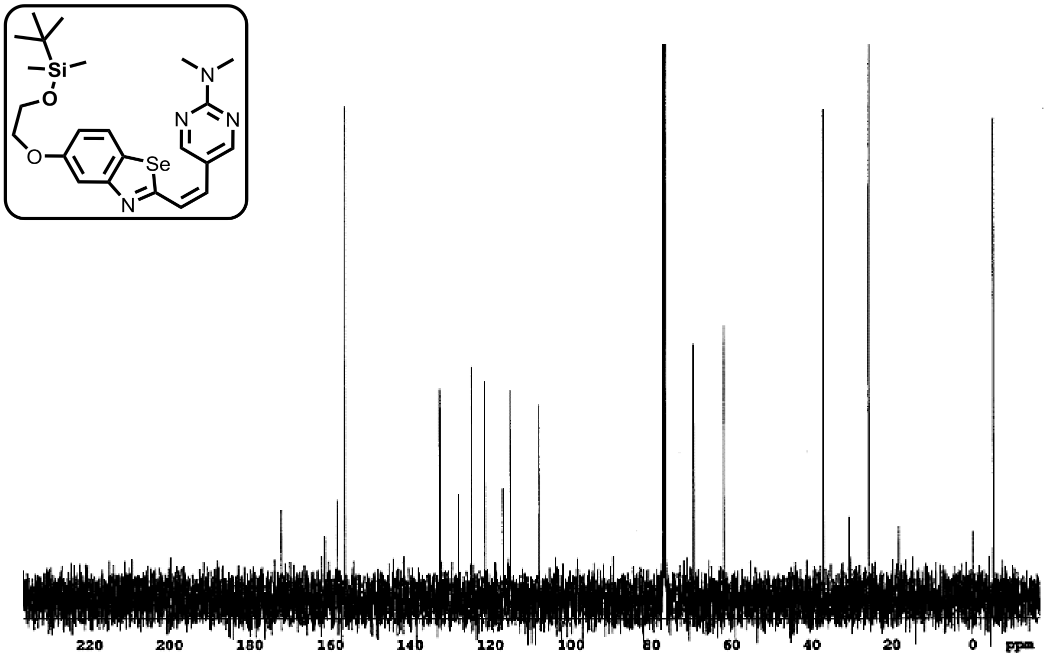

^{13}C NMR Spectrum of compound 6

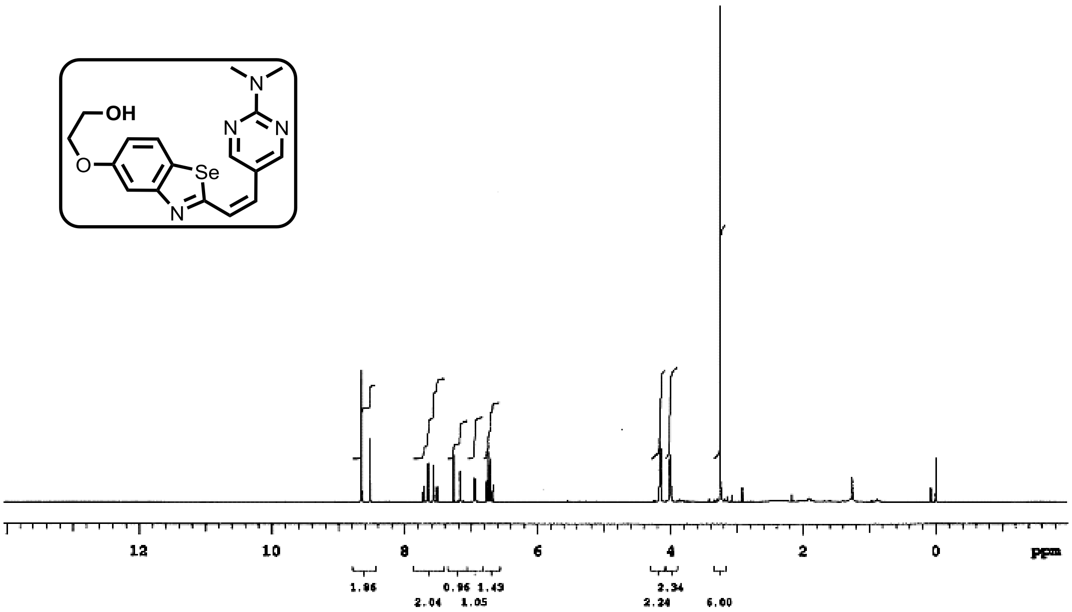

<u>^{1}H spectrum of compound 7</u>

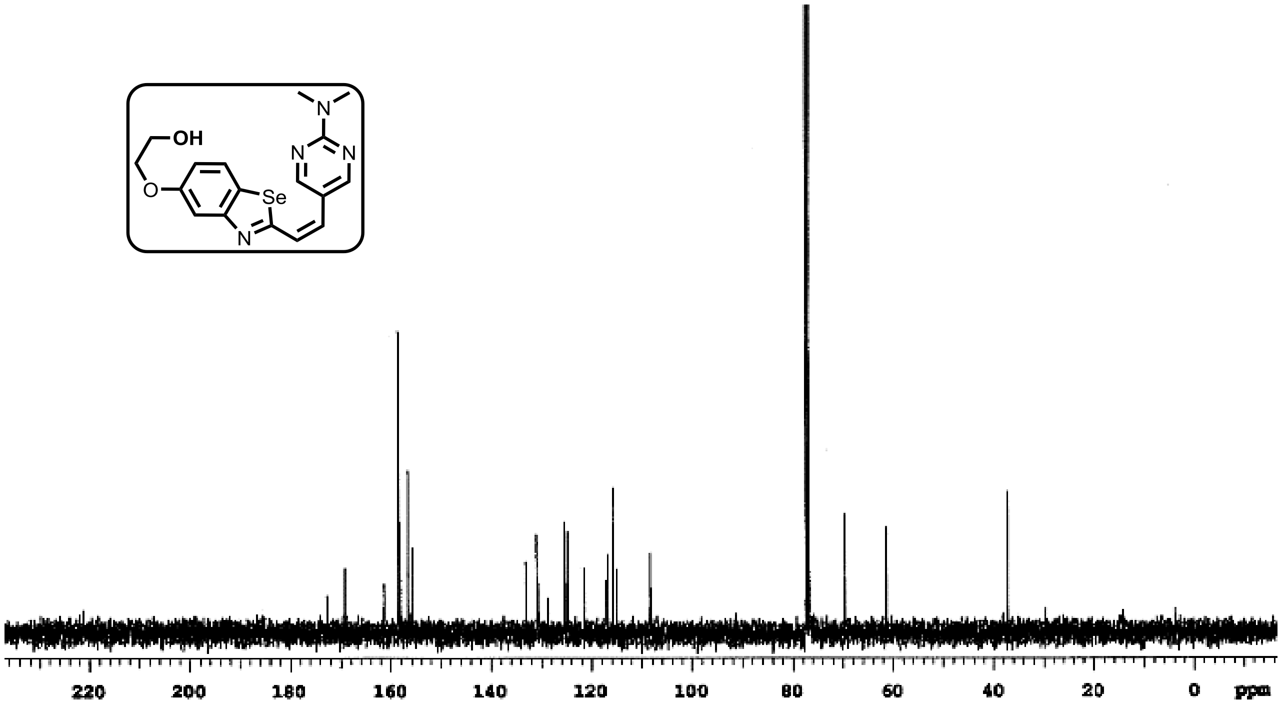

<u>^{13}C NMR Spectrum of compound 7</u>

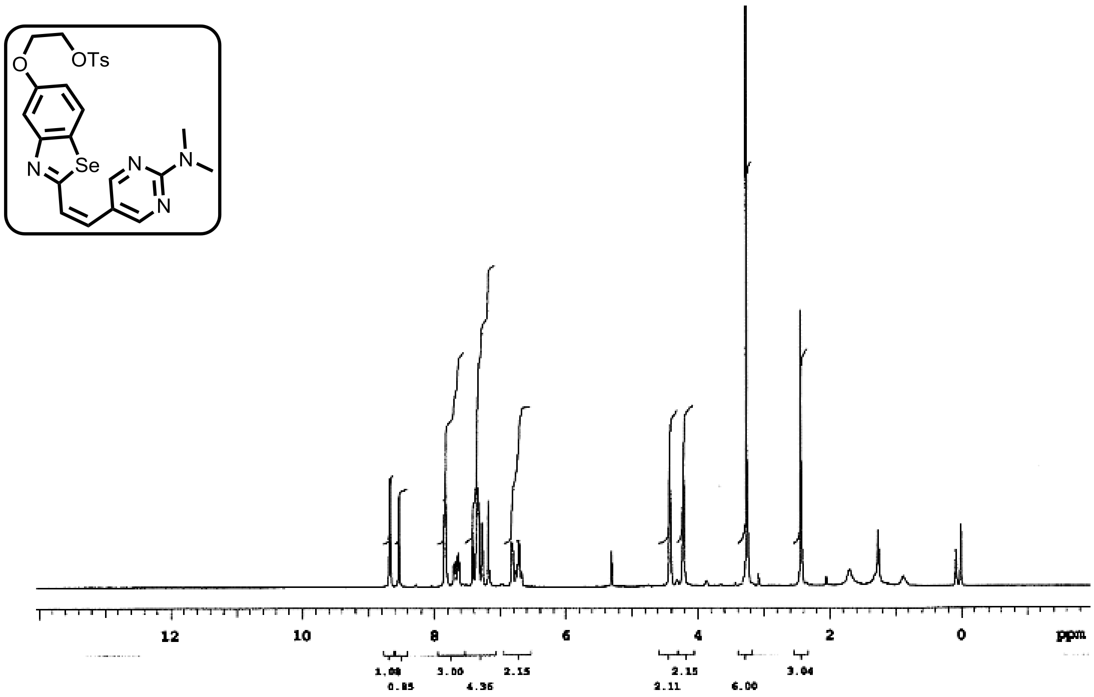

^{1}H spectrum of compound 8

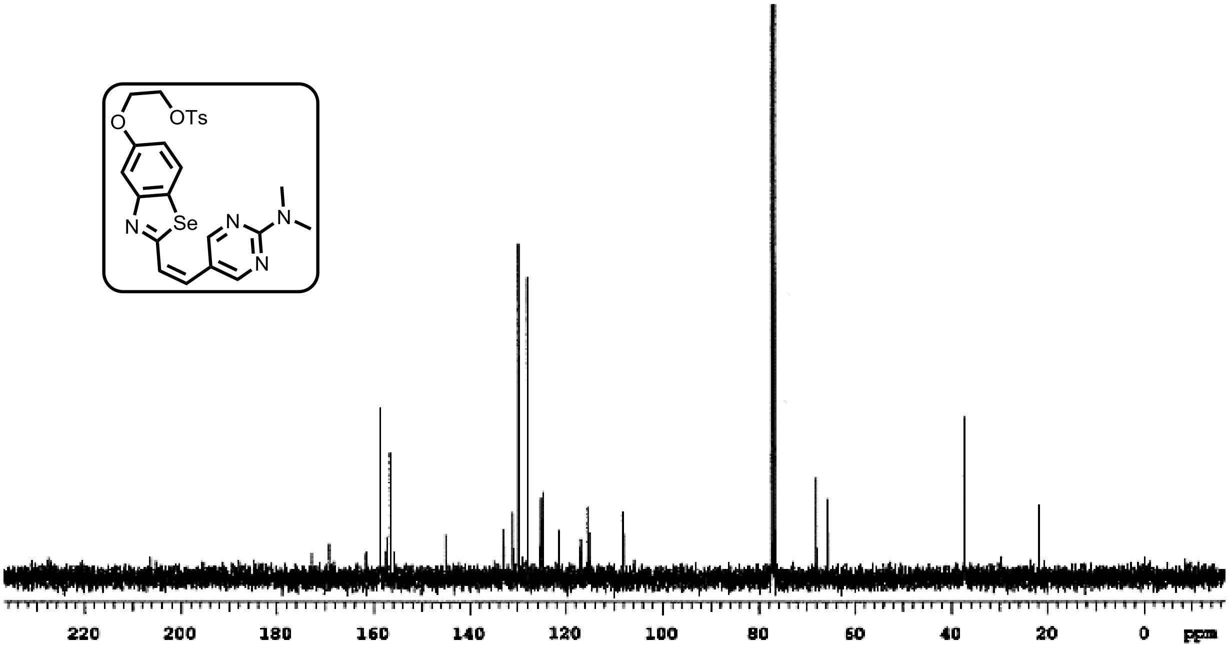

^{13}C NMR Spectrum of compound 8

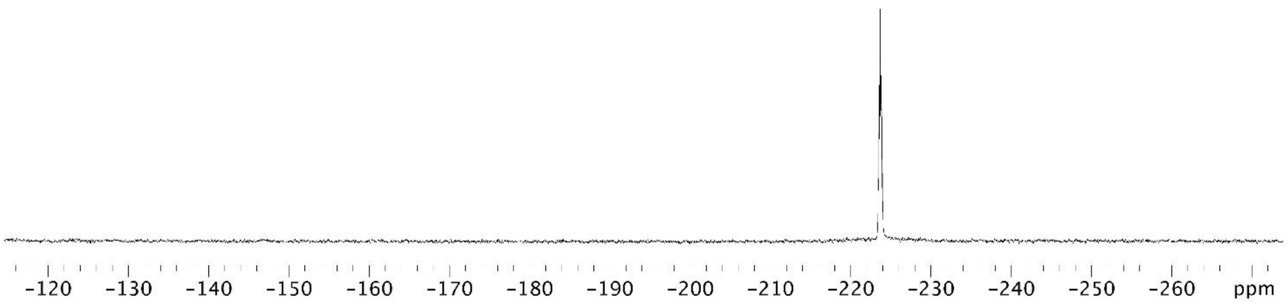

^{19}F NMR Spectrum for 5

References.

1 Yu, K., Park, J. & Yang, S. Synthesis of [^{18}F]Fluorocholine analogues as potential imaging agents for PET studies. *Bull Korean Chem Soc* **25**, 506-510 (2004).

2 Redon, S., Kabri, Y., Crozet, M. & Vanelle, P. One pot preparation of 2-(alkyl)arylbenzoselenazoles from the coressponding N-(acetyl)benzoyl-2-iodoanilines via a microwave assisted methodology. *Tet Lett* **55**, 5052-5054 (2014).

3 Sheldrick, G. APEX II and SAINT. *Bruker Analytical X-Ray Instruments, Inc., Madison, WI* (2012).

4 Sheldrick, G. *Acta Cryst* **A64**, 112-122 (2008).

5 Klunk, W. *et al.* Uncharged thioflavin-T derivatives bind to amyloid-beta protein with high affinity and readily enter the brain. *Life Sci.* **69**, 1471-1484 (2001).

6 Zhuang, Z. *et al.* Structure-activity relationships of imidazo[1,2-a]pyridines as ligands for detecting amyloid plaques in the brain. *J. Med. Chem.* **46**, 237-243 (2003).

7 Cirrito, J. R. *et al.* Serotonin signaling is associated with lower amyloid-beta levels and plaques in transgenic mice and humans. *Proc. Natl. Acad. Sci. U. S. A.* **108**, 14968-14973, doi:10.1073/pnas.1107411108 (2011).

8 Holtzman, D., Bales, K., Tenkova, T., Paul, S. & et.al. Apolipoprotein E isoform-dependent amyloid deposition and neuritic degeneration in a mouse model of Alzheimer's disease. *Proc Natl Acad Sci USA* **97**, 2892-2897 (2000).

9 DeMattos, R., O'dell, M., Parsadanian, M., Holtzman, D. & et.al. Clusterin promotes amyloid plaque formation and is critical for neuritic toxicity in a mouse model of Alzheimer's disease. *Proc Natl Acad Sci USA* **99**, 10843-10848 (2002).

10 Cairns, N. J., Taylor-Reinwald, L. & Morris, J. C. Autopsy consent, brain collection, and standardized neuropathologic assessment of ADNI participants: the essential role of the neuropathology core. *Alzheimer's & dementia : the journal of the Alzheimer's Association* **6**, 274-279, doi:10.1016/j.jalz.2010.03.012 (2010).

11 Bagchi, D. P. *et al.* Binding of the radioligand SIL23 to alpha-synuclein fibrils in Parkinson disease brain tissue establishes feasibility and screening approaches for developing a Parkinson disease imaging agent. *PloS one* **8**, e55031, doi:10.1371/journal.pone.0055031 (2013).

12 Choi, S. R. *et al.* Preclinical properties of 18F-AV-45: a PET agent for Abeta plaques in the brain. *J. Nucl. Med.* **50**, 1887-1894, doi:10.2967/jnumed.109.065284 (2009).

13 Klunk, W. E. *et al.* Binding of the positron emission tomography tracer Pittsburgh compound-B reflects the amount of amyloid-beta in Alzheimer's disease brain but not in transgenic mouse brain. *J. Neurosci.* **25**, 10598-10606 (2005).

14 Sundaram, G. S. *et al.* Synthesis, characterization, and preclinical validation of a PET radiopharmaceutical for interrogating Abeta (beta-amyloid) plaques in Alzheimer's disease. *EJNMMI research* **5**, 112, doi:10.1186/s13550-015-0112-4 (2015).

15 Sivapackiam, J. *et al.* Synthesis, molecular structure, and validation of metalloprobes for assessment of MDR1 P-glycoprotein-mediated functional transport. *Dalton Trans* **39**, 5842-5850 (2010).

16 Yan, P. *et al.* Characterizing the appearance and growth of amyloid plaques in APP/PS1 mice. *J. Neurosci.* **29**, 10706-10714, doi:10.1523/JNEUROSCI.2637-09.2009 (2009).

17 Bero, A. W. *et al.* Neuronal activity regulates the regional vulnerability to amyloid-beta deposition. *Nat. Neurosci.* **14**, 750-756, doi:10.1038/nn.2801 (2011).

18 Sundaram, G. *et al.* Characterization of a brain permeant fluorescent molecule and visualization of Aβ parenchymal plaques, using real-time multiphoton imaging in transgenic mice. *Org. Lett.* **16**, 3640-3643 (2014).
